# Data-Mining Methodology to Improve the Scientific Production Quality in Turkey Meat and Carcass Characterization Studies

**DOI:** 10.3390/ani14142107

**Published:** 2024-07-19

**Authors:** José Ignacio Salgado Pardo, Francisco Javier Navas González, Antonio González Ariza, José Manuel León Jurado, Nuno Carolino, Inês Carolino, Juan Vicente Delgado Bermejo, María Esperanza Camacho Vallejo

**Affiliations:** 1 Department of Genetics, Faculty of Veterinary Sciences, University of Córdoba, 14071 Córdoba, Spain; josalgadopardo@outlook.com (J.I.S.P.); fjng87@hotmail.com (F.J.N.G.); juanviagr218@gmail.com (J.V.D.B.); 2 Agropecuary Provincial Centre, Diputación de Córdoba, 14071 Córdoba, Spain; jomalejur@yahoo.es; 3 Centro de Investigação Vasco da Gama, Escola Universitária Vasco da Gama, 3020-210 Coimbra, Portugal; nuno.carolino@iniav.pt (N.C.); ines.carolino@iniav.pt (I.C.); 4 Instituto Nacional de Investigação Agrária e Veterinária, Polo de Inovação da Fonte Boa—Estação Zootécnica Nacional, 2005-424 Santarém, Portugal; 5 Centro de Investigação Interdisciplinar em Sanidade Animal, Faculdade de Medicina Veterinária, Universidade de Lisboa, 1300-477 Lisboa, Portugal; 6 Laboratório Associado para a Ciência Animal e Veterinária, Faculdade de Medicina Veterinária, Universidade de Lisboa, 1300-477 Lisboa, Portugal; 7 Instituto Superior de Agronomia, Universidade de Lisboa, 1349-017 Lisboa, Portugal; 8 Andalusian Institute of Agricultural and Fisheries Research and Training (IFAPA), Alameda del Obispo, 14004 Córdoba, Spain; mariae.camacho@juntadeandalucia.es

**Keywords:** data-mining, turkey meat quality, physical traits, chemical profile, publication quality traits, biostatistical tool

## Abstract

**Simple Summary:**

Simple Summary: Currently, research on livestock production suffers from isolation from other disciplines and a generalist nature, which makes publishing in top-tier journals a very difficult task. This situation is even more drastic when it comes to turkey meat research, which is an underdeveloped area that has historically suffered from a lack of resources compared to other species. For this reason, the aim of the present study is to develop a tool that allows researchers to determine which carcass and meat quality traits are related to increased interest by the scientific community and the quality standards of the journals in which studies are published. Variables improving journal standards include carcass dressing traits, muscle fibers properties, pH, colorimetry, some texture and water captivity traits, and chemical composition. Contrarily, carcass or piece yield is not a recommended variable to be performed in studies, as this parameter did not show a clear impact on publication quality. Finally, measures after 72 h are contraindicated since they showed a correlation with poor journal quality standards. Thus, this work can be used as a guideline for designing turkey carcass and meat quality studies, describing parameters to prioritize in order to maximize the impact quality of publication in the scientific community.

**Abstract:**

The present research aims to describe how turkey meat and carcass quality traits define the interest of the scientific community through the quality standards of journals in which studies are published. To this end, an analysis of 92 research documents addressing the study of turkey carcass and meat quality over the last 57 years was performed. Meat and carcass quality attributes were dependent variables and included traits related to carcass dressing, muscle fiber, pH, colorimetry, water-holding capacity, texture, and chemical composition. The independent variables comprised publication quality traits, including journal indexation, database, journal impact factor (JIF), quartile, publication area, and JIF percentage. For each dependent variable, a data-mining chi-squared automatic interaction detection (CHAID) decision tree was developed. Carcass or piece yield was the only variable that did not show an impact on the publication quality. Moreover, color and pH measurements taken at 72 h postmortem showed a negative impact on publication interest. On the other hand, variables including water-retaining attributes, colorimetry, pH, chemical composition, and shear force traits stood out among the quality-enhancing variables due to their low inclusion in papers, while high standards improved power.

## 1. Introduction

Increasing advances in research and how these advances are valued by the scientific community suggest the need to evaluate the determinants of the impact of publications on researchers. Thus, the search for new methodological alternatives or statistical strategies that improve the potential scope of research studies is necessary. However, due to the increase in researchers and the diminishment of research funds availability [1], publishing in a top-tier journal has become dramatically difficult in recent years [2]. Research on livestock carries additional difficulties for publishing in high-impact journals [2] because of the multidisciplinary nature of animal research; for example, including medical studies together with basic biology, welfare, and animal care in a wide spectrum of species [2]. All of these subjects are commonly included in a few unspecific knowledge areas, such as ‘Biology’, ‘Animal Science and Zoology’, or ‘Food Science and Technology’. This multidisciplinary nature tends to cause those areas to be underrepresented when searching the literature [3]. Another cluster in which livestock research is commonly found is ‘Veterinary Science’; however, animal production sciences perform a secondary role behind medical studies in this area [4]. The most specific cluster for livestock research might be ‘Agriculture, Dairy, and Animal Science’. However, this cluster includes plant and environmental research alongside livestock, where dairy cows and pigs are the most popular species [5]. Moreover, the agriculture and animal production fields suffer from a lack of bidirectional interdisciplinary co-citations compared to other fields [6], which implies additional difficulties in reaching a high impact factor [7]. With regard to poultry, research has mainly focused on nutrition, production, environment, and reproduction, and the cluster ‘products, processing, and marketing’ has moved into a secondary role [8]. Furthermore, private research interests have hindered poultry research, aiming to develop intellectual property market tools [9,10].

Research in poultry meat quality began after World War II in industrialized countries to satisfy the increasing demand for animal protein [11]. As a consequence of exhaustive growth selection in broilers [9], together with industrialized practices in transport and at slaughter, carcass defects and meat quality irregularities appeared [11,12]. This previous lack of research investment in meat quality was reported by Kempster [13], who attributed it to the great challenge for the meat industry to reach quality standards at an affordable price. These first meat characterizations included color, pH, texture-related traits, and fat and connective tissue contents. Since then, meat quality research has developed to include new attributes such as animal welfare during slaughter or muscle morphology and its influence on meat properties [11].

Food quality could be considered the confluence of consumers’ needs with the intrinsic and extrinsic attributes of a food product [14]. Intrinsic quality properties include sensory properties, shelf life, chemical and nutritional attributes, and health aspects [14], while extrinsic attributes include traits less related to the physical product, such as animal welfare or environmental aspects and marketing features, such as brands, origin, and packaging [15]. Due to the historic lack of measurability of extrinsic quality attributes, only intrinsic ones are addressed in this study.

Measuring meat’s intrinsic qualities in terms of carcass and meat physical characteristics is expensive and has an economic impact on carcasses due to value depreciation caused by sample collection. This has led to a widespread visual carcass grading and classification model and simple measurements of carcass shape or subcutaneous fat cover [13]. Grading for poultry carcass quality, similar to other livestock species, consists of the evaluation of a normal shape that is fully fleshed, meaty, and free of defects [16]. In the poultry industry, carcass grading is optional, contrary to health inspections in other species’ industries, such as bovines and pigs (which is specified in Regulation (EU) No. 1308/2013). Due to this, there is a lack of uniform criteria when evaluating carcass characteristics among markets [17].

Meat’s intrinsic qualities also involve health, nutritional value, and sensory quality traits [14]. Consumers’ growing demand for fat-free animal protein sources [18], together with ‘functional food’ properties [19], offers a great opportunity for the poultry meat industry. This has led to a great change in poultry meat quality research over the last 20 years [20]. From simple measures such as carcass weight, pH, or water-holding capacity, poultry meat research has developed other sophisticated measures for analyzing muscle fibers [21] or the definition of the amino acid profile of a piece of meat [22]. This comes together with the ‘less but better’ strategy, a market trend promoting a reduction in meat consumption while eating high-quality products [23]. Consumers’ interests are changing in favor of integrity and traceability, eating enjoyment, and ethical production systems, and they are willing to pay the extra costs that these ‘exclusive’ and high-quality products have [24]. Future topics considered within product quality research will tend to include animal performance and its relationship with product quality, sensory analysis, healthier and functional animal food products, and products derived from indigenous breeds [25]. This is becoming a reality in the turkey industry, where several papers have recently been published addressing the effect of meat and carcass quality traits on genotype. A recent study defined the most important quality variables differencing breeds [26,27,28,29] and successfully developed a statistical tool for breed traceability [27]. However, there is no literature addressing the relationship between these meat and carcass quality traits and interest in them in the scientific community.

Therefore, the present article aims to use a data-mining analysis to identify the primary traits to include in turkey carcass and meat quality studies to make them have a higher impact on the scientific community. This would be beneficial for optimizing the economic resources of research institutions, especially for low-income enterprises, such as local breed-related studies or alternative production systems.

## 2. Materials and Methods

### 2.1. Literature Search Strategy and Exclusion Criteria

Data collection was performed as described in previous research [20,30,31,32]. The different research studies were sought using two platforms, (www.google.scholar.es and www.sciencedirect.com, (accessed on 10 June 2024), and the search was last performed in July 2024. The selection of these repositories was based on the fact that they offer tools for data extraction to its analysis, while other browsers, such as www.webofscience.com/wos/woscc/basic-search and www.ncbi.nlm.gov/pubmed/ (accessed on 10 June 2024) do not. This fact prompted their exclusion as information sources, as suggested by Iglesias Pastrana, Navas González, Ciani, Barba Capote, and Delgado Bermejo [31] and González Ariza, Navas González, Arando Arbulu, León Jurado, Delgado Bermejo, and Camacho Vallejo [20].

The literature search was performed using the following keywords: ‘Meat/carcass quality/traits’, followed by ‘turkey’, ‘Meleagris gallopavo’, or any term semantically related [33]. A total of 92 documents were collected for the present study. The bibliography used was published in the English language from 1968 until 2024. The included parameters are shown within their cluster and accompanied by the references of the works from which they were collected in Table 1.

Information about the papers was included in the analysis, considering the country and continent of precedence, year of publication, the publishing journal, and the publication quality traits, such as journal indexation (Yes/No), database (Not indexed, JCR (Journal Citation Reports), SJR (Scimago Journal and Country Rank), or Scopus), journal impact factor (JIF), quartile, publication area, and JIF percentage. Journal indexation (indexed), database, quartile, publication area (area), JIF, and JIF percentage were the independent variables used in the posterior analysis.

A total of 1.210 individual observations were recorded considering different carcass cuts from which they were obtained: carcass remainder, breast, complete leg, thigh, drumstick, wings, head, neck, feet, shank, back, heart, liver, giblets, kidney, lungs, spleen, pancreas, gallbladder, proventriculus, gizzard (full and empty), stomach, complete intestine, small intestine, cecum, abdominal fat, fat pad, ovary, oviduct, feathers, skin, feather plus skin, blood, and waste.

The aforementioned 28 meat and carcass attributes included in Table 1 were considered in the statistical analysis as dependent variables. Thus, the presence or absence (Yes/No) of the aforementioned dependent variables in each study was collected and used in the statistical analysis.

Since the techniques and procedures used to collect the measurements were standardized in the studies, there was no need to record the specific methodologies and techniques used to determine each particular explanatory variable. This decision was made because, even if differences may exist among standardized techniques, these might be negligible, as supported by scientific evidence [121,122]. Thus, the inclusion of each measurement was used as classification criteria to elaborate a data-mining chi-squared automatic interaction detection (CHAID) decision tree according to the quality of the journal in which the study was published.

### 2.2. Data Analysis

#### 2.2.1. Data-Mining CHAID Decision Tree

In order to classify, predict, interpret, and discretely categorize data manipulation, the data-mining CHAID decision tree was employed using the classification tree routine of commercial software (SPSS Version 26.0 for Windows, SPSS, Inc., Chicago, IL, USA). For each dependent variable, comprised of the different carcass or meat quality traits, a decision tree was developed. A root node, branches, and leaf nodes are included using the CHAID-based algorithm decision support tool. Each internal node in the tree was built around a publication quality characteristic (input variables), while the so-called pre-pruning process met a significance split criterion of the chi-square test (*p* < 0.05).

According to Breiman et al. [123], pruning must be carried out in such a way that trees do not have a large number of branches and that branches that significantly contribute to the overall fit are not overlooked. When the computation of a tree exhaustively depicts the significant relationships across the detection of independent variables, those nodes not contributing to the overall prediction are discarded. Additionally, the CHAID method penalizes the model’s complexity. For this purpose, the statistical analyses were developed through the Bonferroni inequality significant level adjustment. Moreover, Breiman’s method resembles forward stepwise regression with a reduction in the number of steps using chi-squared, in opposition to F-to-enter-based tests. Each branch is the representation of a result of the test (in a number of two or more), and each leaf node reflects a category level of the target variable. Thus, decisions are made at each nodal point, and each data record continues down through the tree along a path until the record reaches a terminal node [124].

#### 2.2.2. Data-Mining CHAID Decision Tree Reliability: Cross-Validation

Cross-validation was performed once the model was established in order to ensure that the set of significant predictors properly measured the differences between the prediction errors for a tree. It was applied to a new sample and a training sample. Through the use of the complexity parameter and the cross-validated error, cross-validation of the decision tree was performed to define the accuracy of the model when generalized for unseen data. Ten-fold cross-validation was carried out, keeping each individual observation in either the training sample or study data [125]. The resubstitution error rate is employed to measure the proportion of misclassified original observations by various subsets of the original tree. This is performed to determine the shortest tree with the greatest number of significant relationships. On the other hand, the lowest resubstitution rate is not always the optimal choice because this tree will present a bias. Similarly, large trees will introduce random variation into the predictions by over-fitting outliers. For these reasons, X-fold cross-validation is used to obtain a cross-validated error rate rather than selecting a tree based on the resubstitution error rate. X-fold cross-validation involves the creation of a number of X-random subsets of the original data, setting one portion aside as a test set, constructing a tree for the remaining X-1 portions, and evaluating the tree using the test portion. An estimate of the error is evaluated, and this is repeated for all portions. Adding up the error across the X portions represents the cross-validated error rate. The tree exhibiting the lowest cross-validated error rate is selected due to its good data-fitting properties.

## 3. Results

### 3.1. Study Georeferencing

**Figure 1:**
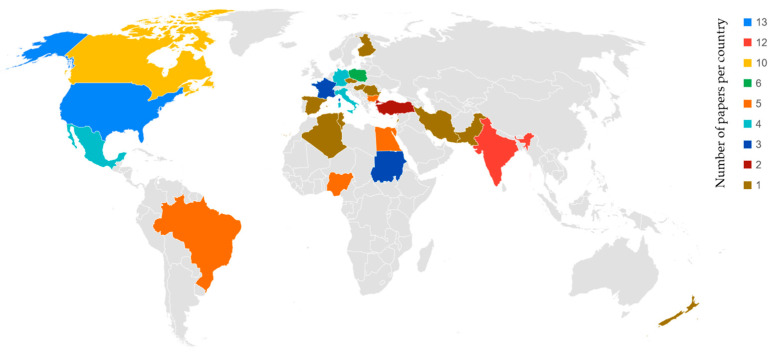
Territorial distribution and number of papers per country.

Figure 1 shows the country distribution of turkey meat and carcass quality studies used in the present research. Most papers included in this research are from the USA (13 studies), India (12 studies), and Canada (10 studies). However, America is the continent that leads the ranking of research study production, followed by Europe, with 32 and 27 studies, respectively. Within Europe, Poland is the country with the most papers (6 studies), followed by Bulgaria (5 studies), and Italy and Germany share the third position, with 4 studies each.

### 3.2. Data-Mining CHAID Decision Tree: Splitting, Pruning, and Building

Supplementary Figure S1 represents the different data-mining CHAID decision trees obtained from the chi-square dissimilarity matrices built in the present study for each dependent variable.

The traits that showed a higher inclusion percentage were carcass/piece yield (71.7%) and slaughter weight (70.5%). However, while slaughter weight showed a moderately positive effect on the journal’s impact factor, carcass and piece yield do not seem to have a positive effect on journal standards.

On the other hand, those traits showing a consistent improver effect on journal standards while being less frequently included were cholesterol (0.6%), cooking loss (0.7%), shear force (4.0%), drip loss (4.6%), pH (5.0%), water-holding capacity (8.2%), ash (10.3%), pH24 (10.7%), b* meat (11.1%), a* meat (11.2%), moisture (11.2%), L* meat (11.4%), protein (12.8%), and fat (12.8%).

### 3.3. Data-Mining CHAID Decision Tree: Splitting, Pruning, and Building

Finally, the robustness and the validity of the obtained results were cross-validated. For this, the number of erroneously classified observations was computed. For each of the trees obtained, the different risk estimates and standard errors computed by applying the cross-validation test did not differ from the results of the model without the cross-validation test. Thus, the stability of the used model was guaranteed. Values for risk estimates and standard errors for methods applied in each tree are shown in Supplementary Table S1.

## 4. Discussion

The present study develops an updated evaluation of international research studies focusing on the inclusion criteria of the different traits analyzed in carcass and meat characterization in the turkey species worldwide. In this aspect, North America, Europe, and Asia are the main sources of studies focused on turkey meat and carcass quality. These same regions overlap with those where the majority of papers in high-standard journals are published [6]. South America, despite being the clade of turkey domestication [126], shows a low rate of publication of papers regarding carcass and meat quality. This could be due to a lack of investment in research compared to high-income countries [127], together with the great cost of meat and carcass quality research [13]. In addition to this, special attention to local breeds has been shown in certain countries such as Mexico, Egypt, Iran, Lebanon, Nigeria, Bulgaria, and Turkey. Native genotypes play an important role in developing countries, where they are mainly reared in backyard farming [20]. On the other hand, research studies in high-income countries are mainly focused on commercial hybrid strains. According to the results obtained in the present study, only 5 of 14 papers (35.7%) based on local genotypes have been published in an indexed journal. These results agree with those described by González Ariza, Navas González, Arando Arbulu, León Jurado, Delgado Bermejo, and Camacho Vallejo [20], which analyzed the variability of meat and carcass quality from worldwide native chicken genotypes and evidenced the limited impact of research involving these populations.

Regarding the traits used in this research, despite no consistent trend in the inclusion of the carcass/piece weight variable in studies (41.7%), its inclusion produced a slight improvement in the impact factor of the journals in which studies were published. The importance of this trait lies in the fact that it allows the estimation of the carcass and its components’ yield [68]. On the other hand, the weight of those primary cuts of the carcass, such as breast, thighs, drumsticks, wings, and back, is considered a parameter of growing interest since the acquisition of the whole carcass is no longer popular among consumers, who show a preference for the aforementioned cuts [128]. Moreover, primary cuts adapted to specified market demands of weight and shape have been described as a crucial descriptor of poultry meat sensory features [11]. On the other hand, carcass/piece yield trait is often included in meat quality articles (71.7%). This could be due to the ability to reflect the total amount of meat that is put on the market after the removal of viscera and offal from the carcass [128]. Furthermore, the estimation of the carcass components’ yield is a valuable source of information for producers and intermediaries as long as each primary cut has a different market value [12,71,129]. Hence, knowledge of carcass components’ yield could be applied to each genotype selection program targeting those higher-valued cuts [130]. In addition, carcass dressing is a particularly interesting trait in turkeys, as it is the poultry species with the highest carcass dressing percentages [131]. However, its inclusion did not show a clear effect on the standard of journals, according to the results of the present study. This could suggest that this parameter is a basic carcass attribute included in every kind of study.

Research studies have shown a low tendency to include the cold carcass weight trait in turkey meat quality studies (14.0%), even though its presence is correlated with studies of greater interest for high-impact journals. This could be due to the weight of the cold carcass being widely measured in the meat industry to control evaporative losses during carcass refrigeration [132], which can reach 1–3% of carcass weight in turkeys [90]. However, the slaughter body weight explains 95% of the variation observed in the cold carcass weight trait [101]. This fact might explain the low percentage of inclusion of this variable in carcass characterization studies.

The slaughter weight variable showed a high tendency of inclusion in studies (70.5%) and has a moderate positive correlation to the journal’s impact factor. It is widely used in the turkey industry as a grading factor in modern hybrid strains, distinguishing a heavy, medium-heavy, and medium type [131]. The high use of this variable in the studies allows identifying carcass dressing percentage [68]. Moreover, slaughter weight has also been described to have implications in carcass components’ proportions and dressing percentages [128], as well as intrinsic meat quality traits [11,80].

Even though including muscle fiber diameter has been shown to improve the journals’ interest degree in the studies, muscle fiber characteristics have not been as deeply studied meat quality parameters in poultry as in other livestock species, despite turkey displaying similar meat quality traits [34] and muscle abnormalities as some mammals [17,133]. Furthermore, fiber morphology could be used as a meat quality and animal welfare indicator as soon as it has been correlated to several muscle pathologies [134]. This could cause a change in the journal’s attention to this parameter in turkey meat quality studies, performing as a journal standard improver. On the other hand, muscle growth occurs in turkeys through an increase in the muscle fiber diameter and not by hyperplasia, as in many other species [34]. This fact may explain the reason for the inclusion of muscle density parameters in some studies.

pH measurements are widely spread through meat quality studies. Moreover, the inclusion of pH at slaughter and 24 h postmortem has been reported to significantly improve the journal’s impact factor percentile (JIF %). Meat acidification after slaughter has been described to regulate proteolysis and postmortem muscle contraction, playing an important role in meat tenderness [14,135]. As a major indicator of postmortem biochemical changes, pH has been reported to be correlated to other meat characteristics such as color, drip loss, tenderness, and juiciness [13]. pH is also an indicator of PSE (pale-soft-exudative) meat alterations, which have been described to occur in 40% of turkey breasts [11]. Moreover, early pH measurements have shown a correlation with the technological quality of meat processing in terms of drip loss and product shelf life [80]. On the other hand, pH measurements taken 24 h postmortem are commonly used to draw pH decline curves due to their impact on meat processing [80]. However, pH measurements at 72 h are barely included in papers (0.2%) and show a negative correlation with the journal quartile in which studies are published. This could be because the pH decline in poultry is reached just a few hours postmortem [11,14,136]. Despite this time being influenced by breed, slow-growing turkey breeds have been reported to reach 4 times longer than high-performance strains, in which acidification processes have been described to take only 4 h [136].

The inclusion of color measurements (L*, a*, b* coordinates of CIELAB color space) was strongly positively correlated with the journal’s impact factor. Meat and skin color traits are the first quality attributes noticed by the consumer at the time of purchase, hence their importance [14,128]. Consumers tend to prefer poultry meat that has a color similar to what they are used to, demanding white/pink meat [137] and penalizing extreme paleness and darkness [138]. Furthermore, a growing market niche demands yellowness in poultry meat [139] due to an association with freshness and free-range and natural feed systems by the consumer [140]. Meat color has also been associated with muscle alterations in poultry slaughterhouses. Unlike chicken, heat stress and excitation cause pale meat, similar to those PSE issues in pork, instead of reddish carcasses [17,141]. In addition, meat color is an interesting parameter for meat quality studies because of its relationship with pH, water-holding capacity, cooking loss, and other textural properties [11,136]. Previous authors reported that lighter meat samples (higher L* values) are negatively correlated with water-holding capacity in turkeys [142]. Nevertheless, a* and b* values have not been described to have a significant correlation with any physical meat quality parameter [136], except for the white striping pathology [12]. Contrarily, color measurements (L*, a*, and b*) taken at 72 h postmortem showed a correlation with lower journal quality standards in which studies had been published when including this trait. This could be due to a lack of useful information on turkey meat color from 24 h postmortem, as described by Alvarado and Sams [141]. Thus, color and pH measurements taken at 72 h postmortem are not recommended parameters to include in meat turkey quality studies since they do not offer valuable information nor improve the quality standards of the journal where they are published. Similar findings were described by González Ariza, Navas González, Arando Arbulu, León Jurado, Delgado Bermejo, and Camacho Vallejo [20], who recommended the exclusion of those measurements in fowl meat and carcass quality studies, attaching it to a lack of representativity when prior sampling moments had been considered.

Consumers rank palatability and tenderness as the most important attributes of meat eating qualities. However, these traits are the major source of consumer complaints as well [14]. Complaints about turkey meat toughness during the 1970s stimulated research investments to improve this property of this species [12,136]. Nevertheless, the numerous factors influencing meat tenderness and their interrelations have challenged research in this area, where little progress has been made [14]. In this sense, due to the known role of connective tissue in meat toughness, collagen content and solubility have been the most often studied meat tenderness indicators [143]. However, results in the present research show a tendency not to include collagen analysis in turkey meat quality research studies (0.2%). Moreover, its inclusion has evidenced a slight correlation with the journal’s quality standards in which the studies are published. This could be due to the lower importance of hardness or toughness problems in poultry meat in comparison with beef or pork [34]. Furthermore, intramuscular collagen analysis in turkey breast showed a good level of collagen cross-linking and maturation [12] and has been proven to share characteristics with chicken [129] despite the huge difference in age at slaughter. The shear force has a low rate of inclusion in turkey meat quality studies (4.0%) despite having performed as an interest enhancer for higher journal impact factors. Traditionally, the instrumental measurement of shear force by texture analyzers has been one of the most used methods to determine meat tenderness [144]. It has been employed as an alternative to very time-consuming and costly taste panels [145], which historically have been complemented and correlated with meat chemical composition studies [146]. However, shear force estimation shows limitations, as its correlation to meat tenderness depends largely on the muscle, its genetic basis, and production system [14].

Despite tenderness being the most-valued meat quality attribute, flavor and juiciness are considered increasingly important traits [14]. Juiciness is a harder parameter to quantify and is sometimes combined with tenderness [14]. Additionally, juiciness is assessed from a function involving water-holding capacity and cooking loss [128]. However, when trying to develop models to predict sensory texture attributes based on instrumental measurements, a consensus is not common [147]. The inclusion of water-holding capacity has improved the journal’s impact factor in which studies were published. This is in line with literature describing this attribute as the most significant economic trait in meat [13]. However, since the late 1980s, there has been a notable lack of a simple and cost-effective technique for its implementation in slaughterhouses. That might be why, in recent studies, water-holding capacity has been estimated by assessing other meat quality parameters, such as color and pH [72,136,148]. On the other hand, drip loss and cooking loss have shown a low tendency of inclusion in papers (4.6 and 7.0%, respectively) despite their improving effect on the studies’ JIF percentile. Drip loss has been pointed out as one of the main parameters considered by both producers and consumers to give added value to meat products [149]. Moreover, the drip loss trait has been considered a good determiner of water-holding capacity, being the most affected parameter in PSE meat [150], and keeps correlation to the L* value [149], ultimate pH, and cooking loss [150]. Cooking loss, on its side, has an inverse relationship with water-holding capacity [128] and negatively affects both tenderness and juiciness [151]. Due to the water evaporation while cooking and the dripping of water and fat, the leanness of turkey meat and its often skinless cooking make it an important meat quality parameter to determine [151].

Springiness (0.2%), chewiness (0.2%), gumminess (0.2%), and fragmentation index (0.5%) have scarcely been measured in observations. However, they showed a moderate enhancer effect on the journal quality in which papers were published. The employment of springiness, chewiness, and gumminess in meat quality studies has been controversial. Previous authors have reported that these traits are not well correlated to their descriptive texture [20,152,153] and are imprecise in predicting sensory analysis [154]. However, an investigation has shown their possible role as discriminators of meat quality [147]. Contrarily, the fragmentation index has been described as a useful parameter to provide information about toughness and tenderness when comparing different turkey-rearing systems [153].

Although there is a slight tendency for papers not to include the crude chemical components (moisture, proteins, fats, ash, and cholesterol), their inclusion has shown a correlation with improving the studies’ attractiveness by journals with higher quality criteria. Moisture is a determinant factor in meat quality analysis due to its high influence on meat tenderness, juiciness, firmness, appearance, and economic value [155]. Meat proteins have historically been in the spotlight of research since meat has been historically considered a high-value protein source [14]. Moreover, turkey meat has an especially high protein composition, rounding 28%, against 14–18% in other poultry meat [156]. On the other hand, fat composition has been deeply monitored because of its impact on health [18,19]. However, the selection for leanness in poultry meat has led to a negative impact on meat texture attributes since the collagen content of muscles in turkeys with different growth rates is highly variable. Slow-growing birds show a higher collagen content than fast-growing ones [11]. This may be mainly attributed to the lower cross-sectional area of muscle fibers, leading to a higher content of endomysial collagen relative to muscle volume [157]. Turkey carcasses have a high lean content and relatively low fat content, with muscle inclusion ranging between 2 and 5%, which has made it a desirable source of nutrients for consumers who wish to eat healthy food [22,131,156]. Additionally, turkey meat is characterized by a high mineral content that is necessary for satisfying the normal function of many organs of the human body [158]. Finally, dietary cholesterol has become a special interest from both the scientific and consumer perspectives, in order to determine its impact on health. Consumers associate meat as a source of cholesterol with a high implication for obesity, coronary diseases, and cancer [22]. In this sense, turkey could be a slice of particularly interesting meat because it has the lowest level of cholesterol in comparison with other types of meat [156].

## 5. Conclusions

Conclusively, the present research can be used as a guide for the comprehensive evaluation of literature resources that determine which methodology and meat quality parameters should be used in turkey meat quality studies when aiming to publish papers that are of great interest to the scientific community. There is a general tendency to conduct simple studies, but it has been evidenced that as studies add more quality traits and, therefore, become more complex, they are published in journals with higher research quality values. Results show that North America, Europe, and Asia showed a higher level in the scope of research since these regions have historically made a greater economic effort in the development of research studies based on the nutritional quality of turkey-derived products. Despite being highly recorded and included in observations, carcass/piece yield (%) did not exhibit a clear effect on journals’ standards, while carcass/piece weight (kg), despite not being as widely included, showed a consistent effect on journal quality. The close relationship between cold carcass weight and slaughter weight shows that the first trait has shown a low percentage of inclusion in research studies so far despite being an improver of studies’ interest. Among the lowest-included traits with a strong positive effect on standards were cooking loss, shear force, drip loss, pH (at slaughter and after 24 h), water-holding capacity, meat colorimetry (L*, a*, and b* meat), moisture, protein, fat, ash, and cholesterol. On the other hand, results obtained in the present research suggest that some measurements (pH and color traits) taken at 72 h postmortem can be avoided due to the negative influence on the impact of the research. Hence, this study develops a tool to tailor and improve research efficiency while also maximizing the efficacy of economic funding, which is especially useful for low-income initiatives such as local genotypes research.

## Figures and Tables

**Table 1:** Cluster of the traits considered in the studies.

Cluster	Trait	
Carcass dressing traits	Carcass/piece weight	[22,28,29,34,35,36,37,38,39,40,41,42,43,44,45,46,47,48,49,50,51,52,53,54,55,56,57,58,59,60,61,62,63,64,65,66,67,68,69,70,71,72,73,74,75,76,77,78,79,80,81,82,83,84,85,86,87,88,89,90,91,92,93,94,95,96,97,98,99,100,101,102,103]
	Carcass/piece yield	
	Cold carcass weight	
	Slaughter weight	
Muscle fiber properties	Muscle fiber diameter	[34]
pH	pH	[22,28,29,34,38,46,47,57,58,59,60,62,70,71,72,73,79,80,82,83,84,85,86,87,88,90,92,95,96,97,98,99,102,103,104,105,106,107,108,109,110,111,112,113,114,115,116,117,118]
	pH 24 h	
	pH 72 h	
Color-related traits	L* meat	[22,28,29,34,37,38,40,46,47,57,58,60,62,70,71,73,79,80,82,86,87,88,90,92,95,96,97,99,102,104,105,108,110,111,115,116,117,118,119,120]
	a* meat	
	b* meat	
	L* meat 72 h	
	a* meat 72 h	
	b* meat 72 h	
Water-holding capacity	Water-holding capacity	[28,29,34,38,40,46,47,48,50,56,57,58,59,60,62,71,73,79,82,83,85,86,87,88,90,92,95,96,98,99,102,103,104,105,106,107,109,110,111,112,113,114,115,117,118,119,120]
	Drip loss	
	Cooking loss	
Texture-related traits	Shear force	[28,29,34,46,56,57,60,71,79,82,83,86,87,90,99,103,105,106,107,110,112,114,115]
	Springiness	
	Gumminess	
	Chewiness	
	Fragmentation index	
Chemical composition	Moisture	[29,34,37,38,45,46,54,56,58,59,60,61,62,65,69,71,79,84,85,86,87,90,92,96,98,102,104,105,106,109,111,112,114,115,118]
	Protein	
	Fat	
	Ash	
	Collagen	
	Cholesterol	

## Data Availability

The data used to support the findings of this study can be made available by the corresponding author upon request.

## Supplementary Materials

The following supporting information can be downloaded at https://www.mdpi.com/article/10.3390/ani14142107/s1: Figure S1: Graphical representation of the CHAID decision tree about the inclusion of each meat and carcass quality trait in the studies, considering the publication quality trait as the clustering criterion. (A) carcass/piece weight, (B) carcass/piece yield, (C) cold carcass weight, (D) slaughter weight, (E) muscle fiber diameter, (F) pH, (G) pH 24 h, (H) pH 72 h, (I) L* meat, (J) a* meat, (K) b* meat, (L) L* meat 72 h, (M) a* meat 72 h, (N) b* meat 72 h, (O) drip loss, (P) water-holding capacity, (Q) cooking loss, (R) shear force, (S) springiness, (T) gumminess, (U) chewiness, (V) fragmentation index, (W) moisture, (X) protein, (Y) fat, (Z) ash, (AA) collagen, (AB) cholesterol; (1) Included, (2) Not included. Table S1: Values for risk estimates and standard errors for both methods applied in the cross-validation test for each tree.

## References

[1] Rabesandratana T. (2015). Plan for EU research funds raises ire. Science.

[2] Krauskopf E., Garcia F., Funk R. (2017). Bibliometric analysis of multi-language veterinary journals. Transinformação.

[3] Fang H. (2015). Classifying research articles in multidisciplinary sciences journals into subject categories. KO Knowl. Organ..

[4] Choudhary D., Vadlamudi V., Singh U. (2017). Research Output of Veterinary and Animal Sciences as Seen from the Indian Veterinary Journal. Libr. Waves.

[5] Rodriguez-Ledesma A., Cobo M.J., Lopez-Pujalte C., Herrera-Viedma E. (2015). An overview of animal science research 1945–2011 through science mapping analysis. J. Anim. Breed. Genet..

[6] Christopher M.M., Marusic A. (2013). Geographic trends in research output and citations in veterinary medicine: Insight into global research capacity, species specialization, and interdisciplinary relationships. BMC Vet. Res..

[7] Christopher M.M. (2015). Weighing the impact (factor) of publishing in veterinary journals. J. Vet. Cardiol..

[8] Vaziri E., Maghsoudi A., Feizabadi M., Faraji-Arough H., Rokouei M. (2022). Scientometric evaluation of 100-year history of Poultry Science (1921–2020). Poult. Sci..

[9] Council N.R. (2015). Critical Role of Animal Science Research in Food Security and Sustainability.

[10] Hoffmann I. (2005). Research and investment in poultry genetic resources–challenges and options for sustainable use. World’s Poult. Sci. J..

[11] Grashorn M. (2010). Research into poultry meat quality. Br. Poult. Sci..

[12] Zampiga M., Soglia F., Baldi G., Petracci M., Strasburg G.M., Sirri F. (2020). Muscle abnormalities and meat quality consequences in modern turkey hybrids. Front. Physiol..

[13] Kempster A. (1989). Carcass and meat quality research to meet market needs. Anim. Sci..

[14] Hocquette J.-F., Richardson R.I., Prache S., Medale F., Duffy G., Scollan N.D. (2005). The future trends for research on quality and safety of animal products. Ital. J. Anim. Sci..

[15] Bernabéu R., Rabadán A., El Orche N.E., Díaz M. (2018). Influence of quality labels on the formation of preferences of lamb meat consumers. A Spanish case study. Meat Sci..

[16] Park B. (2016). Quality Evaluation of Poultry Carcass. Computer Vision Technology for Food Quality Evaluation.

[17] Pérez-Chabela M.L., Totosaus A. (2006). Poultry carcass evaluation. Handb. Food Sci. Technol. Eng..

[18] Zhu R., Fogelholm M., Jalo E., Poppitt S.D., Silvestre M.P., Møller G., Huttunen-Lenz M., Stratton G., Sundvall J., Macdonald I.A. (2022). Animal-based food choice and associations with long-term weight maintenance and metabolic health after a large and rapid weight loss: The PREVIEW study. Clin. Nutr..

[19] Cavani C., Petracci M., Trocino A., Xiccato G. (2009). Advances in research on poultry and rabbit meat quality. Ital. J. Anim. Sci..

[20] González Ariza A., Navas González F.J., Arando Arbulu A., León Jurado J.M., Delgado Bermejo J.V., Camacho Vallejo M.E. (2022). Variability of Meat and Carcass Quality from Worldwide Native Chicken Breeds. Foods.

[21] Weng K., Huo W., Li Y., Zhang Y., Zhang Y., Chen G., Xu Q. (2022). Fiber characteristics and meat quality of different muscular tissues from slow-and fast-growing broilers. Poult. Sci..

[22] Gálvez F., Domínguez R., Pateiro M., Carballo J., Tomasevic I., Lorenzo J.M. (2018). Effect of gender on breast and thigh turkey meat quality. Br. Poult. Sci..

[23] Pais D.F., Marques A.C., Fuinhas J.A. (2020). Reducing meat consumption to mitigate climate change and promote health: But is it good for the economy?. Environ. Model. Assess..

[24] Napolitano F., Girolami A., Braghieri A. (2010). Consumer liking and willingness to pay for high welfare animal-based products. Trends Food Sci. Technol..

[25] Lorenzo J.M. (2020). Grand Challenges in Product Quality. Front. Anim. Sci..

[26] Salgado Pardo J.I., Navas González F.J., González Ariza A., León Jurado J.M., Galán Luque I., Delgado Bermejo J.V., Camacho Vallejo M.E. (2023). Study of meat and carcass quality-related traits in Turkey populations through discriminant canonical analysis. Foods.

[27] Salgado Pardo J.I., González Ariza A., Navas González F.J., León Jurado J.M., Díaz Ruiz E., Delgado Bermejo J.V., Camacho Vallejo M.E. (2024). Discriminant canonical analysis as a tool for genotype traceability testing based on turkey meat and carcass traits. Front. Vet. Sci..

[28] Hiscock H.M., Leishman E.M., Vanderhout R.J., Adams S.M., Mohr J., Wood B.J., Baes C.F., Barbut S. (2022). Describing the relationships among meat quality traits in domestic turkey (*Meleagris gallopavo*) populations. Poult. Sci..

[29] Zampiga M., Tavaniello S., Soglia F., Petracci M., Mazzoni M., Maiorano G., Meluzzi A., Clavenzani P., Sirri F. (2019). Comparison of 2 commercial turkey hybrids: Productivity, occurrence of breast myopathies, and meat quality properties. Poult. Sci..

[30] González Ariza A., Navas González F.J., León Jurado J.M., Arando Arbulu A., Delgado Bermejo J.V., Camacho Vallejo M.E. (2022). Data mining as a tool to infer chicken carcass and meat cut quality from autochthonous genotypes. Animals.

[31] Iglesias Pastrana C., Navas González F.J., Ciani E., Barba Capote C.J., Delgado Bermejo J.V. (2020). Effect of research impact on emerging camel husbandry, welfare and social-related awareness. Animals.

[32] McLean A.K., Gonzalez F.J.N. (2018). Can scientists influence donkey welfare? Historical perspective and a contemporary view. J. Equine Vet. Sci..

[33] Schlosser R.W., Wendt O., Bhavnani S., Nail-Chiwetalu B. (2006). Use of information-seeking strategies for developing systematic reviews and engaging in evidence-based practice: The application of traditional and comprehensive Pearl Growing. A review. Int. J. Lang. Commun. Disord..

[34] Werner C., Riegel J., Wicke M. (2008). Slaughter performance of four different turkey strains, with special focus on the muscle fiber structure and the meat quality of the breast muscle. Poult. Sci..

[35] Farghly M., Alagawany M., Abd El-Hack M. (2018). Feeding time can alleviate negative effects of heat stress on performance, meat quality and health status of turkey. Br. Poult. Sci..

[36] Ojewola G., Abasiekong S., Nwachukwu C. (2001). Methionine supplementation in the productive efficiency, carcass characteristics and economics of growing indigenous turkey. Niger. J. Anim. Sci..

[37] Roberson K., Rahn A., Balander R., Orth M., Smith D., Booren B., Booren A., Osburn W., Fulton R. (2003). Evaluation of the growth potential, carcass components and meat quality characteristics of three commercial strains of tom turkeys. J. Appl. Poult. Res..

[38] Laudadio V., Introna M., Lastella N.M., Tufarelli V. (2014). Feeding of low-fibre sunflower (*Helianthus annus* L.) meal as substitute of soybean meal in turkey rations: Effects on growth performance and meat quality. J. Poult. Sci..

[39] Gibril S., Hassan H.A., Yassin O.E., Shamseldin R.M. (2013). Growth Performance and Carcass Characteristics of Turkeys (*Meleagris gallopavo*) under semi intensive System in the Sudan. Univ. Khartoum J. Agric. Sci..

[40] Ferket P., Malheiros R., Moraes V., Ayoola A., Barasch I., Toomer O., Torrent J. (2020). Effects of functional oils on the growth, carcass and meat characteristics, and intestinal morphology of commercial turkey toms. Poult. Sci..

[41] Anandh M.A. (2019). Effect of sex on slaughter and carcass characteristics of broad breasted bronze turkeys (*Meleagris gallopavo*). Asian J. Anim. Sci..

[42] Shamseldin R., Gibril S., Atta M., Yassin O., Hassan A. (2014). Effect of rearing system, slaughter age and sex on turkey (*Meleagris gallopavo*) carcass components percentages. Res. Opin. Anim. Vet. Sci..

[43] Ribarski S., Oblakova M., Miteva D., Oblakov N. (2015). Comparative study of carcass characteristics and chemical composition of meat in north caucasian bronze and wild turkey (*Meleagris gallopavo silvestris Vieillot*). Sci. Technol..

[44] Case L., Wood B., Miller S. (2012). The investigation of ultrasound technology to measure breast muscle depth as a correlated trait to breast meat yield in turkey (*Meleagris gallopavo*). J. Anim. Sci..

[45] Przywitowski M., Mikulski D., Zdunczyk Z., Rogiewicz A., Jankowski J. (2016). The effect of dietary high-tannin and low-tannin faba bean (*Vicia faba* L.) on the growth performance, carcass traits and breast meat characteristics of finisher turkeys. Anim. Feed. Sci. Technol..

[46] Śmiecińska K., Hnatyk N., Daszkiewicz T., Kubiak D., Matusevičiu P. (2015). The effect of frozen storage on the quality of vacuum-packaged turkey meat. Vet. Ir. Zootech..

[47] Oblakova M., Hristakieva P., Mincheva N., Ivanova I., Lalev M., Ivanov N., Penchev I. (2021). Effect of dietary herbal essential oils on the performance and meat quality of female turkeys broilers. Trakia J. Sci..

[48] Ribarski S., Oblakova M. (2016). Slaughter yield and quality of meat from wild turkey (*Meleagris gallopavo silvestris Vieillot*) reared in hunting reserve in South Bulgaria. Trakia J. Sci..

[49] Ojewola G., Ukachukwu S., Onyenucheya F. (2000). Comparative carcass characteristics of indigenous turkey poults fed different agro-industrial by-products. Niger. J. Anim. Sci..

[50] Safiyu K.K., Sogunle O.M., Egbeyale L.T., Shittu T.A. (2019). An exploratory study on the effects of rearing system and plumage colour on performance, carcass characteristics and meat quality of local turkeys. Int. J. Health Anim. Sci. Food Saf..

[51] Moran E., Leeson S., Summers J. (1978). Large turkey commercial strain comparisons of performance, carcass quality and meat yield during 1969 and 1977. Can. J. Anim. Sci..

[52] Lemme A., Frackenpohl U., Petri A., Meyer H. (2004). Effects of reduced dietary protein concentrations with amino acid supplementation on performance and carcass quality in turkey toms 14 to 140 days of age. Int. J. Poult. Sci..

[53] Salmon R. (1984). Effect of grower and finisher protein on performance, carcass grade, and meat yield of turkey broilers. Poult. Sci..

[54] Sell J., Ferket P., Angel C., Scheideler S., Escribano F., Zatari I. (1989). Performance and carcass characteristics of turkey toms as influenced by dietary protein and metabolizable energy. Nutr. Rep. Int..

[55] Moran E., Summers J., Orr H. (1969). The effect of absolute alterations in energy concentration of developing and finishing diets for the Large White Turkey on performance and carcass quality with a note on the correlation of back skin fat and grade of finish. Br. Poult. Sci..

[56] Majumdar S., Bhanja S., Singh R., Agarwal S. (2005). Effect of age on the carcass traits and meat quality of turkey poults. J. Appl. Anim. Res..

[57] Boukhris H., Damergi C., Najar T., Samet A. (2017). Transport stress impact on postmortem metabolisms of turkey meat quality. J. New Sci..

[58] Drażbo A., Kozłowski K., Ognik K., Zaworska A., Jankowski J. (2019). The effect of raw and fermented rapeseed cake on growth performance, carcass traits, and breast meat quality in turkey. Poult. Sci..

[59] Mikulski D., Jankowski J., Zdunczyk Z., Wróblewska M., Sartowska K., Majewska T. (2009). The effect of selenium source on performance, carcass traits, oxidative status of the organism, and meat quality of turkeys. J. Anim. Feed. Sci..

[60] Mikulski D., Jankowski J., Zdunczyk Z., Juskiewicz J., Slominski B. (2012). The effect of different dietary levels of rapeseed meal on growth performance, carcass traits, and meat quality in turkeys. Poult. Sci..

[61] Işgüzar E. (2003). Growth, carcass traits and meat quality of Bronze and White turkeys in Isparta province of Turkey. Arch. Anim. Breed..

[62] Hristakieva P., Oblakova M., Mincheva N., Ivanova I., Lalev M., Ivanov N., Penchev I. (2021). Effect Of Dry Herbal Feed Additive On The Performance And Meat Quality Of Turkeys Broilers. J. Hyg. Eng. Des..

[63] Anandh M.A., Jagatheesan P.N.R. Effect of Sex on Slaughter and Carcass Traits of Beltsville Small White Turkey (Meleagris gallopavo) under Indian Hot Humid Climatic Condition. 2017.

[64] Anna Anandh M. (2017). Effect of rearing systems on slaughter and carcass characteristics of turkey (*Meleagris gallopavo*). Res. J. Anim. Husb. Dairy Sci..

[65] Sell J. (1993). Influence of metabolizable energy feeding sequence and dietary protein on performance and selected carcass traits of tom turkeys. Poult. Sci..

[66] Darshana B., Bhaisare D.T., Churchil R.R., Punniamurthy N. (2014). Effect of dietary supplementation of herbal seeds on carcass traits of turkey poults. Vet. World.

[67] Leeson S., Caston L. (1991). Response of two strains of turkey hens to various protein and energy feeding programs. Poult. Sci..

[68] Taha N., Farran M. (2009). Comparative study of thigh muscles and bones conformation and some carcass traits of local vs. imported turkey strain. Int. J. Poult. Sci..

[69] Melnychuk V., Robinson F., Renema R., Hardin R., Emmerson D., Bagley L. (1997). Carcass traits and reproductive development at the onset of lay in two lines of female turkeys. Poult. Sci..

[70] Le Bihan-Duval É., Berri C., Baéza É., Santé V., Astruc T., Rémignon H., Le Pottier G., Bentley J., Beaumont C., Fernandez X. (2003). Genetic parameters of meat technological quality traits in a grand-parental commercial line of turkey. Genet. Sel. Evol..

[71] Cavalcanti É.N.F., Giampietro-Ganeco A., Mello J.L., Fidelis H.A., Oliveira R.F., Pereira M.R., Villegas-Cayllahua E.A., Souza R.A., Souza P.A., Borba H. (2021). Breast meat quality of turkey breeder hens at disposal age affected by deep pectoral myopathy. Poult. Sci..

[72] Barbut S. (1993). Colour measurements for evaluating the pale soft exudative (PSE) occurrence in turkey meat. Food Res. Int..

[73] Wojtysiak D., Górska M. (2018). Effect of aging time on meat quality and rate of desmin and dystrophin degradation of pale, soft, exudative (PSE) and normal turkey breast muscle. Folia Biol..

[74] Dorra T.I., Ibrahim S.E., Zayed S.M. (2012). Effect of dietary betaine supplementation on growth performance and carcass traits of growing turkey. J. Anim. Poult. Prod..

[75] Biswas A., Mandal A., Singh R. (2015). Effect of dietary supplementation of chromium picolinate on productive performance, egg quality and carcass traits in laying turkeys. Anim. Nutr. Feed Technol..

[76] Sirohi R., Shukla P.K., Bhattacharyya A., Singh Y., Singh D.N., Kumar A. (2018). Effect of Photoperiod on the Production Performance and Carcass Quality Traits of Turkey Poults. J. Anim. Res..

[77] Biswas A., Divya S., Mandal A., Majumdar S., Singh R. (2014). Effects of dietary supplementation of organic chromium (picolinate) on physical and biochemical characteristics of semen and carcass traits of male turkeys. Anim. Reprod. Sci..

[78] Oke F.O., Onasanya G.O., Adedire A.O., Oduguwa O.O., Obadire S.O., Osofowora A.O. (2014). Effects of feed probiotics on serum biochemistry and carcass characteristics of tropically bred exotic turkey. IOSR J. Agric. Vet. Sci. (IOSR-JAVS).

[79] Ngoka D.A., Froning G., Lowry S., Babji A. (1982). Effects of sex, age, preslaughter factors, and holding conditions on the quality characteristics and chemical composition of turkey breast muscles. Poult. Sci..

[80] Fernandez X., Santé V., Baéza E., Lebihan-Duval E., Berri C., Rémignon H., Babilé R., Pottier G.L.L., Astruc T. (2002). Effects of the rate of muscle post mortem pH fall on the technological quality of turkey meat. Br. Poult. Sci..

[81] Ciurescu G., Vasilachi A., Grosu H. (2020). Efficacy of microbial phytase on growth performance, carcass traits, bone mineralization, and blood biochemistry parameters in broiler turkeys fed raw chickpea (*Cicer arietinum L*., cv. Burnas) diets. J. Appl. Poult. Res..

[82] Parteca S., Tonial I.B., do Prado N.V., da Trindade Alfaro A. (2020). Electrical stunning parameters: Impact on the quality of turkey meat (*Meleagris gallopavo*). J. Food Sci. Technol..

[83] Ylä-Ajos M., Tuominen S., Hänninen L., Ruusunen M., Puolanne E., Valros A. (2012). Gas composition in controlled atmosphere stunning affects turkey meat quality traits. Br. Poult. Sci..

[84] Laudadio V., Tufarelli V., Dario M., D’emilio F., Vicenti A. (2009). Growth performance and carcass characteristics of female turkeys as affected by feeding programs. Poult. Sci..

[85] Farghly M., Abou-Kassem D. (2014). Impacts of feed color and form on growth performance of local turkey. Egypt. J. Nutr. Feed..

[86] Lee H., Erasmus M., Swanson J., Hong H., Kang I. (2016). Improvement of turkey breast meat quality and cooked gel functionality using hot-boning, quarter sectioning, crust-freeze-air-chilling and cold-batter-mincing technologies. Poult. Sci..

[87] Abdel-Ghany A. (2015). The Effect of Origanum majorana Supplementation on Growth Performance, Blood parameters and Meat Quality in BUT9 Commercial Turkeys. J. Anim. Poult. Fish. Prod..

[88] Carvalho R.H.d., Soares A.L., Guarnieri P.D., Oba A., Ida E.I., Shimokomaki M. (2018). Turkey meat. Seasonal effect on meat quality and on dead on arrival index in a commercial plant. Braz. Arch. Biol. Technol..

[89] Javid A., Hussain A., Ashraf M., Mahmud A., Altaf M., Hussain S.M., Bukhari S.M. (2019). Variations in carcass yield and meat sensory quality attributes between turkeys (*Meleagris gallopavo*) reared in free-range and confinement rearing systems. Indian. J. Anim. Res..

[90] Saggin R.F., Prado N.V.d., Dos Santos M.M., Balbinot-Alfaro E., da Trindade Alfaro A. (2022). Air chilling of Turkey carcasses: Process efficiency and impact in the meat quality traits. J. Food Sci. Technol..

[91] Abdel-Kafy E.M., Zayed S., Behiry F.M., Gorgy M., Ahmed M.A., Ibraheim S.E. (2022). Assessment of growth, carcass traits, and some physiological parameters of bronze, and white turkeys (*Meleagris gallopavo*), and their Crosses. Egypt. Poult. Sci. J..

[92] Portillo-Salgado R., Herrera-Haro J.G., Bautista-Ortega J., Ramírez-Bribiesca J.E., Flota-Bañuelos C., Chay-Canul A.J., Cigarroa-Vázquez F.A. (2023). Carcass composition and physicochemical and sensory attributes of breast and leg meat from native Mexican guajolote (*Meleagris g. gallopavo*) as influenced by sex. Arch. Anim. Breed..

[93] Tůmová E., Gous R.M., Chodová D., Ketta M. Differences in growth and carcass composition of growing male and female turkeys. 2020, 65, 330–336.

[94] Sadoudi A., Ait-Kaki A., Bellik Y., Touazi L., Yahi K., Iguer-Ouada M., Hornick J.-L., Moula N. (2024). Effect of olive leaf incorporation in animal feed on broiler turkey (*Meleagris gallopavo*) growth performance, welfare, oxidative status, and blood and biochemical serum parameters. Arch. Anim. Breed..

[95] Yusuff A., Saheed K., Badmos A., DeCampos J., Ajao B., Aremu J. (2021). Effect of plumage colour on carcass characteristics and meat quality of Nigeria local turkeys. Niger. J. Anim. Sci..

[96] Portillo-Salgado R., Herrera-Haro J.G., Bautista-Ortega J., Chay-Canul A.J., Efrén Ramírez-Bribiesca J., Ortega-Cerrilla M.E., Flota-Bañuelos C., Cigarroa-Vázquez F.A. (2022). Effects of slaughter age and gender on carcase characteristics and meat quality of native Mexican Turkey (*M. g. gallopavo*) reared under an extensive production system. Ital. J. Anim. Sci..

[97] Vanderhout R.J., Leishman E.M., Abdalla E.A., Barbut S., Wood B.J., Baes C.F. (2022). Genetic parameters of white striping and meat quality traits indicative of pale, soft, exudative meat in Turkeys (*Meleagris gallopavo*). Front. Genet..

[98] Ghosh S., Saha M. (2023). Growth performance and meat quality of turkey birds produced by the small-holders in south 24 parganas district of west Bengal, India. Explor. Anim. Med. Res..

[99] Leishman E.M., Vanderhout R.J., van Staaveren N., Barbut S., Mohr J., Wood B.J., Baes C.F. (2022). Influence of post mortem muscle activity on turkey meat quality. Front. Vet. Sci..

[100] Pezeshkian Z., Mirhoseini S.Z., Ghovvati S., Ebrahimie E. (2023). Phenotypic evaluation of feed efficiency, growth and carcass traits in native turkeys. J. Cent. Eur. Agric..

[101] Portillo-Salgado R., Herrera-Haro J., Bautista-Ortega J., Chay-Canul A., Ramírez-Bribiesca J., Ortega-Cerrilla M. (2022). Predictive Equations of Carcass Characteristics and Primal Cut Weights of Native Mexican Guajolotes Using Body Measurements. Braz. J. Poult. Sci..

[102] Portillo-Salgado R., Herrera-Haro J.G., Bautista-Ortega J., Cigarroa-Vázquez F.A. (2023). Relationships between technological and nutritional meat quality traits in native Mexican *Meleagris gallopavo gallopavo* L. Agro Product..

[103] Updike M., Zerby H., Sawdy J., Lilburn M., Kaletunc G., Wick M. (2005). Turkey breast meat functionality differences among turkeys selected for body weight and/or breast yield. Meat Sci..

[104] Oblakova M., Ribarski S., Oblakov N., Hristakieva P. (2016). Chemical composition and quality of turkey-broiler meat from crosses of layer light (ll) and meat heavy (mh) turkey. Trakia J. Sci..

[105] Maki A., Froning G. (1987). Effect of post-mortem electrical stimulation on quality of turkey meat. Poult. Sci..

[106] Anandh M.A., Jagatheesan P.R. (2020). Meat quality characteristics of Beltsville Small White broiler and spent hen turkeys (*Meleagris gallopavo*). Int. J. Chem. Stud..

[107] Obanor F., Morton J., Geesink G., Bickerstaffe R. (2005). Effect of processing on turkey meat quality and proteolysis. Poult. Sci..

[108] Blacha I., Krischek C., Klein G. (2014). Influence of modified atmosphere packaging on meat quality parameters of turkey breast muscles. J. Food Prot..

[109] Babji A., Froning G., Ngoka D. (1982). The effect of preslaughter environmental temperature in the presence of electrolyte treatment on turkey meat quality. Poult. Sci..

[110] Northcutt J., Buhr R., Young L. (1998). Influence of preslaughter stunning on turkey breast muscle quality. Poult. Sci..

[111] Bianchi M., Capozzi F., Cremonini M.A., Laghi L., Petracci M., Placucci G., Cavani C. (2004). Influence of the season on the relationships between NMR transverse relaxation data and water-holding capacity of turkey breast meat. J. Sci. Food Agric..

[112] Anandh M.A. Effect of rearing systems on meat quality characteristics of beltsville small white Turkey (Meleagris gallopavo) meat. 2020, 8, 1491–1494. 8.

[113] Patterson B., Matarneh S., Stufft K., Preisser R., Shi H., Gerrard D., England E., Scheffler T., Stewart E., Eilert S. (2017). Pectoralis major muscle of turkey displays divergent function as correlated with meat quality. Poult. Sci..

[114] Babji A., Froning G., Ngoka D. (1982). The effect of preslaughter dietary electrolyte treatment on carcass yield and turkey meat quality characteristics. Poult. Sci..

[115] Heincinger M., Balogh K., Mézes M., Fébel H. (2012). Effects of distillers dried grain with soluble (DDGS) on meat quality, lipid peroxide and some of antioxidant status parameters of fattening turkey. J. Poult. Sci..

[116] Deus D., Kehrenberg C., Schaudien D., Klein G., Krischek C. (2017). Effect of a nano-silver coating on the quality of fresh turkey meat during storage after modified atmosphere or vacuum packaging. Poult. Sci..

[117] Owens C., Sams A. (2000). The influence of transportation on turkey meat quality. Poult. Sci..

[118] Sarica M., Ocak N., Turhan S., Kop C., Yamak U. (2011). Evaluation of meat quality from 3 turkey genotypes reared with or without outdoor access. Poult. Sci..

[119] Sante V., Le Pottier G., Astruc T., Mouchoniere M., Fernandez X. (2000). Effect of stunning current frequency on carcass downgrading and meat quality of turkey. Poult. Sci..

[120] Feng X., Moon S.H., Lee H.Y., Ahn D.U. (2017). Effect of irradiation on the parameters that influence quality characteristics of raw turkey breast meat. Radiat. Phys. Chem..

[121] Arantes-Pereira L., Vargas F.C., Balieiro J.C., Bittante A.M.Q., Sobral P.J. (2016). Reproducibility and correlation between meat shear force measurements by Warner-Bratzler machine and a Texturometer. Int. J. Food Stud..

[122] Lorenzen C., Calkins C., Green M., Miller R., Morgan J., Wasser B. (2010). Efficacy of performing Warner–Bratzler and slice shear force on the same beef steak following rapid cooking. Meat Sci..

[123] Breiman L., Friedman J., Stone C.J., Olshen R.A. (1984). Classification and Regression Trees.

[124] Ceylan Z., Gürsev S., Bulkan S. (2018). An application of data mining in individual pension savings and investment system. Avrupa Bilim. Ve Teknol. Derg..

[125] Baykara B. (2015). Impact of Evaluation Methods on Decision Tree Accuracy. Master’s Thesis.

[126] Thornton E.K., Emery K.F. (2017). The uncertain origins of Mesoamerican turkey domestication. J. Archaeol. Method Theory.

[127] Gertzell E., Magnusson U., Ikwap K., Dione M., Lindström L., Eliasson-Selling L., Jacobson M. (2021). Animal health beyond the single disease approach–A role for veterinary herd health management in low-income countries?. Res. Vet. Sci..

[128] Akinwumi A., Odunsi A., Omojola A., Akande T., Rafiu T. (2013). Evaluation of carcass, organ and organoleptic properties of spent layers of different poultry types. Botsw. J. Agric. Appl. Sci..

[129] Voutila L., Ruusunen M., Jouppila K., Puolanne E. (2009). Thermal properties of connective tissue in breast and leg muscles of chickens and turkeys. J. Sci. Food Agric..

[130] Case L., Miller S., Wood B. (2010). Determination of the optimum slaughter weight to maximize gross profit in a turkey production system. Can. J. Anim. Sci..

[131] Murawska D. (2017). The effect of age on growth performance and carcass quality parameters in different poultry species. Poult. Sci..

[132] James C., Vincent C., de Andrade Lima T., James S. (2006). The primary chilling of poultry carcasses—A review. Int. J. Refrig..

[133] Chan J.T., Omana D.A., Betti M. (2011). Application of high pressure processing to improve the functional properties of pale, soft, and exudative (PSE)-like turkey meat. Innov. Food Sci. Emerg. Technol..

[134] Velleman S., Anderson J., Coy C., Nestor K. (2003). Effect of selection for growth rate on muscle damage during turkey breast muscle development. Poult. Sci..

[135] Zhang X., Owens C.M., Schilling M.W. (2017). Meat: The edible flesh from mammals only or does it include poultry, fish, and seafood?. Anim. Front..

[136] Barbut S. (1997). Occurrence of pale soft exudative meat in mature turkey hens. Br. Poult. Sci..

[137] Wideman N., O’bryan C., Crandall P. (2016). Factors affecting poultry meat colour and consumer preferences-A review. World’s Poult. Sci. J..

[138] Fletcher D. (1999). Poultry meat colour. Poult. Meat Sci. CABI Publ. NY.

[139] Ripoll G., Panea B. (2019). The effect of consumer involvement in light lamb meat on behavior, sensory perception, and health-related concerns. Nutrients.

[140] Hernandez J. (2005). Sensory perception of quality of products across Europe: A case study on poultry quality. Sens. Eval. More Than Just Food.

[141] Alvarado C., Sams A. (2002). The role of carcass chilling rate in the development of pale, exudative turkey pectoralis. Poult. Sci..

[142] McCurdy R., Barbut S., Quinton M. (1996). Seasonal effect on pale soft exudative (PSE) occurrence in young turkey breast meat. Food Res. Int..

[143] Mccormick R.J. (1999). Extracellular modifications to muscle collagen: Implications for meat quality. Poult. Sci..

[144] Thielke S., Lhafi S., Kühne M. (2005). Effects of aging prior to freezing on poultry meat tenderness. Poult. Sci..

[145] Van Oeckel M., Warnants N., Boucqué C.V. (1999). Pork tenderness estimation by taste panel, Warner–Bratzler shear force and on-line methods. Meat Sci..

[146] Prieto N., Roehe R., Lavín P., Batten G., Andrés S. (2009). Application of near infrared reflectance spectroscopy to predict meat and meat products quality: A review. Meat Sci..

[147] Luckett C.R., Kuttappan V.A., Johnson L.G., Owens C.M., Seo H.S. (2014). Comparison of three instrumental methods for predicting sensory texture attributes of poultry deli meat. J. Sens. Stud..

[148] Zhang L., Barbut S. (2005). Rheological characteristics of fresh and frozen PSE, normal and DFD chicken breast meat. Br. Poult. Sci..

[149] Çelen M.F., Söğüt B., Zorba Ö., Demirulus H., Tekeli A. (2016). Comparison of normal and PSE turkey breast meat for chemical composition, pH, color, myoglobin, and drip loss. Rev. Bras. Zootec..

[150] Przybylski W., Sałek P., Kozłowska L., Jaworska D., Stańczuk J. (2022). Metabolomic analysis indicates that higher drip loss may be related to the production of methylglyoxal as a by-product of glycolysis. Poult. Sci..

[151] Küçüközet A.O., Uslu M.K. (2018). Cooking loss, tenderness, and sensory evaluation of chicken meat roasted after wrapping with edible films. Food Sci. Technol. Int..

[152] Meullenet J.F., Gross J. (1999). Instrumental single and double compression tests to predict sensory texture characteristics of foods. J. Text. Stud..

[153] Yalçın M.Y., Şeker M. (2016). Effect of salt and moisture content reduction on physical and microbiological properties of salted, pressed and freeze dried turkey meat. LWT-Food Sci. Technol..

[154] Di Monaco R., Cavella S., Masi P. (2008). Predicting sensory cohesiveness, hardness and springiness of solid foods from instrumental measurements. J. Text. Stud..

[155] Ahamed Rifath F.J. (2021). The Quality Determination of Broiler Chicken Thawed Using Different Techniques.

[156] Okuskhanova E., Rebezov M., Yessimbekov Z., Suychinov A., Semenova N., Rebezov Y., Gorelik O., Zinina O. (2017). Study of water binding capacity, ph, chemical composition and microstructure of livestock meat and poultry. Annu. Res. Rev. Biol..

[157] Fernandez X., Sante V., Baéza E., Lebihan-Duval E., Berri C., Rémignon H., Babile R., Le Pottier G., Millet N., Berge P. (2001). Post mortem muscle metabolism and meat quality in three genetic types of turkey. Br. Poult. Sci..

[158] Amirkhanov K., Igenbayev A., Nurgazezova A., Okuskhanova E., Kassymov S., Muslimova N., Yessimbekov Z. (2017). Research article comparative analysis of red and white Turkey meat quality. Pak. J. Nutr..

