# Supplementary material for: Data-Mining Methodology to Improve the Scientific Production Quality in Turkey Meat and Carcass Characterization Studies
Source: Animals (Basel). 2024 Jul 19;14(14):2107. doi: 10.3390/ani14142107 (PMC11273658; doi:10.3390/ani14142107)
Supplement: Supplementary file 1 [file animals-14-02107-s001.zip › animals-3088178-supplementary/Supplementary Figure S1.pdf]

Supplementary Figure S1. Graphical representation of the CHAID decision tree about the inclusion of each meat and carcass quality trait in the studies, considering the publication quality trait as the clustering criterion. (A) carcass/piece weight, (B) carcass/piece yield, (C) cold carcass weight, (D) slaughter weight, (E) muscle fiber diameter, (F) pH, (G) pH 24h, (H) pH 72h, (I)L* meat, (J) a* meat, (K) b* meat, (L) L* meat 72h, (M) a* meat 72h, (N) b* meat 72h, (O) drip loss, (P) water-holding capacity, (Q) cooking loss, (R) shear force, (S) springiness, (T) gumminess, (U) chewiness, (V) fragmentation index, (W) moisture, (X) protein, (Y) fat, (Z) ash, (AA) collagen, (AB) cholesterol; (1) Included, (2) Not included.

A

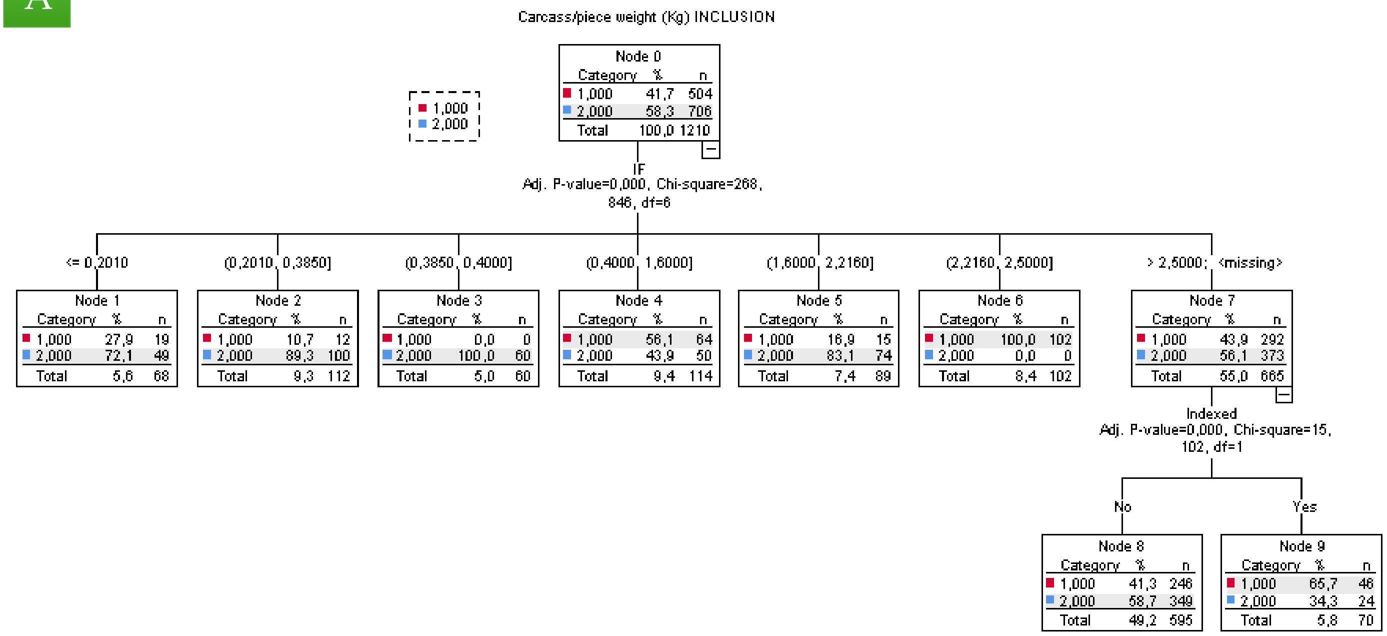

B

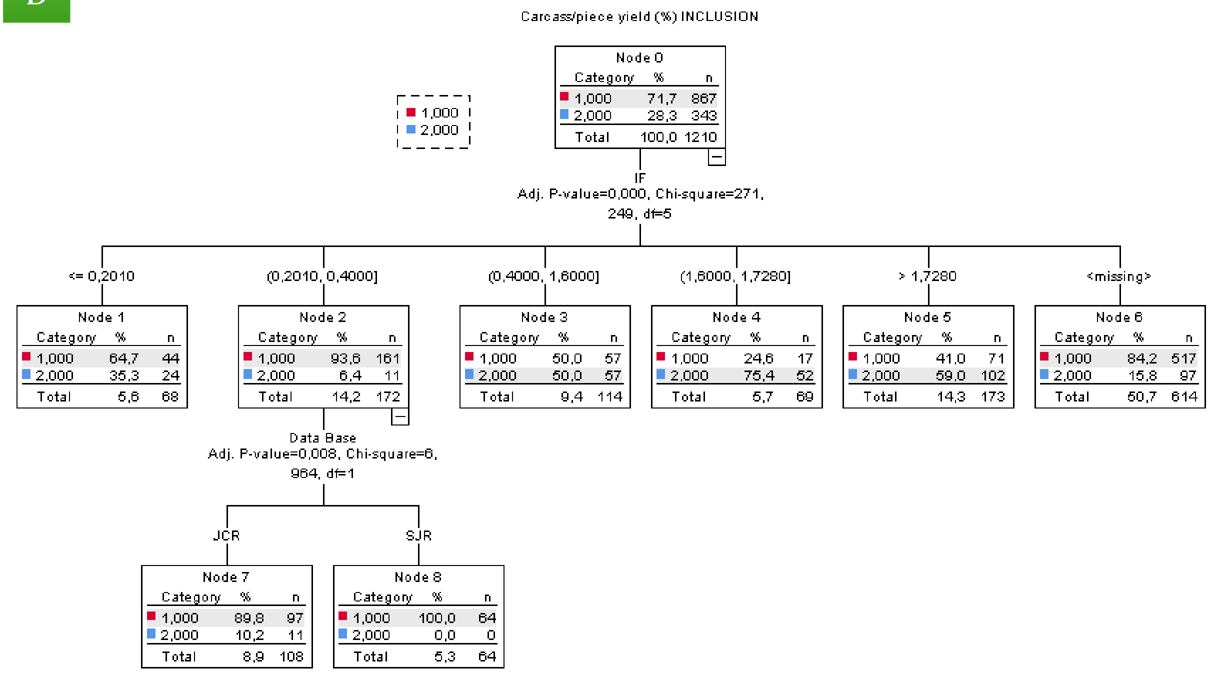

C

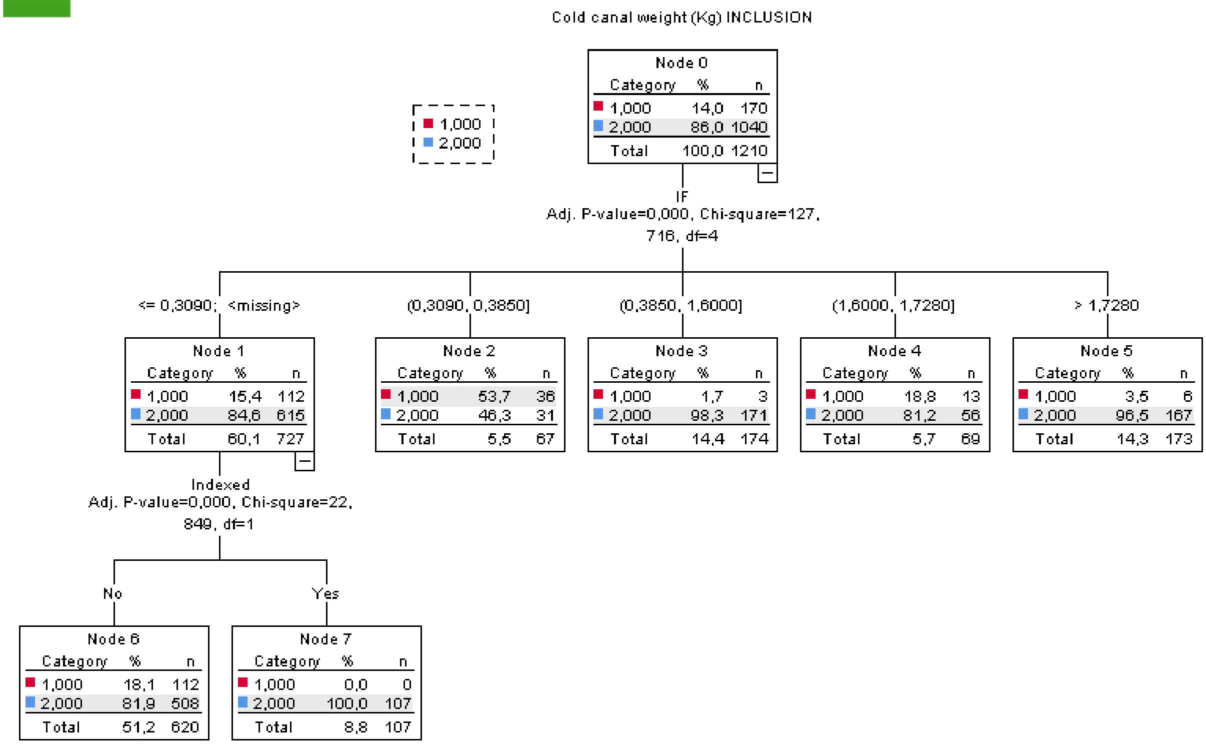
Cold canal weight (Kg) INCLUSION
1,000
2,000
Node 0
Category % n
1,000 14,0 170
2,000 86,0 1040
Total 100,0 1210
IF
Adj. P-value=0,000, Chi-square=127,
716, df=4
<= 0,3090; <missing>
(0,3090, 0,3850]
(0,3850, 1,6000]
(1,6000, 1,7280]
> 1,7280
Node 1
Category % n
1,000 15,4 112
2,000 84,6 615
Total 60,1 727
Node 2
Category % n
1,000 53,7 36
2,000 46,3 31
Total 5,5 67
Node 3
Category % n
1,000 1,7 3
2,000 98,3 171
Total 14,4 174
Node 4
Category % n
1,000 18,8 13
2,000 81,2 56
Total 5,7 69
Node 5
Category % n
1,000 3,5 6
2,000 96,5 167
Total 14,3 173
Indexed
Adj. P-value=0,000, Chi-square=22,
849, df=1
No
Yes
Node 6
Category % n
1,000 18,1 112
2,000 81,9 508
Total 51,2 620
Node 7
Category % n
1,000 0,0 0
2,000 100,0 107
Total 8,8 107

D

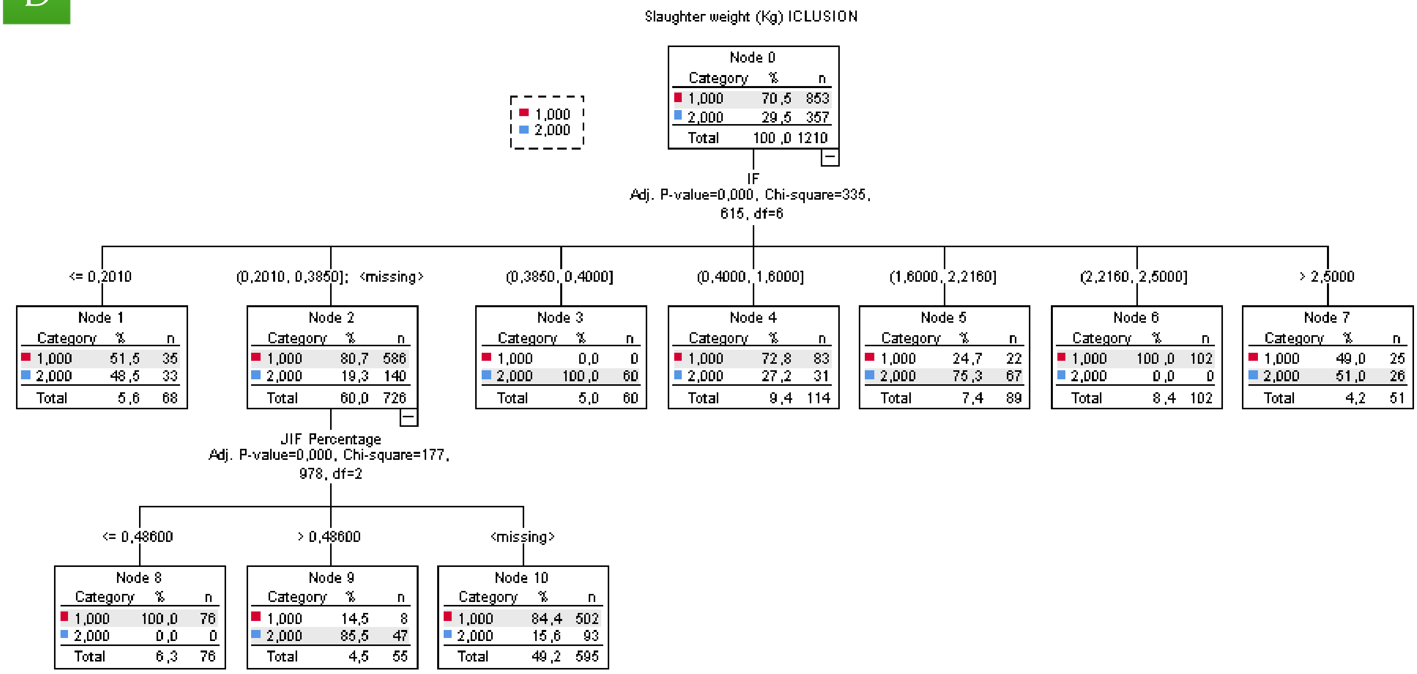
Slaughter weight (Kg) ICLUSION
1,000
2,000
Node 0
Category % n
1,000 70,5 853
2,000 29,5 357
Total 100,0 1210
IF
Adj. P-value=0,000, Chi-square=335,
615, df=6
<= 0,2010
(0,2010, 0,3850]; <missing>
(0,3850, 0,4000]
(0,4000, 1,6000]
(1,6000, 2,2160]
(2,2160, 2,5000]
> 2,5000
Node 1
Category % n
1,000 51,5 35
2,000 48,5 33
Total 5,6 68
Node 2
Category % n
1,000 80,7 586
2,000 19,3 140
Total 60,0 726
Node 3
Category % n
1,000 0,0 0
2,000 100,0 60
Total 5,0 60
Node 4
Category % n
1,000 72,8 83
2,000 27,2 31
Total 9,4 114
Node 5
Category % n
1,000 24,7 22
2,000 75,3 67
Total 7,4 89
Node 6
Category % n
1,000 100,0 102
2,000 0,0 0
Total 8,4 102
Node 7
Category % n
1,000 49,0 25
2,000 51,0 26
Total 4,2 51
JIF Percentage
Adj. P-value=0,000, Chi-square=177,
978, df=2
<= 0,48600
> 0,48600
<missing>
Node 8
Category % n
1,000 100,0 76
2,000 0,0 0
Total 6,3 76
Node 9
Category % n
1,000 14,5 8
2,000 85,5 47
Total 4,5 55
Node 10
Category % n
1,000 84,4 502
2,000 15,6 93
Total 49,2 595

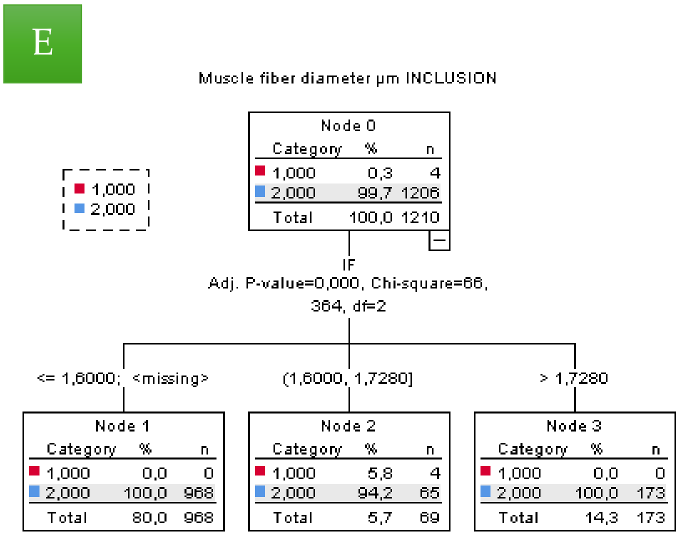
E
Muscle fiber diameter μm INCLUSION
1,000
2,000
Node 0
Category % n
1,000 0,3 4
2,000 99,7 1206
Total 100,0 1210
IF
Adj. P-value=0,000, Chi-square=66,
364, df=2
<= 1,6000; <missing>
(1,6000, 1,7280]
> 1,7280
Node 1
Category % n
1,000 0,0 0
2,000 100,0 968
Total 80,0 968
Node 2
Category % n
1,000 5,8 4
2,000 94,2 65
Total 5,7 69
Node 3
Category % n
1,000 0,0 0
2,000 100,0 173
Total 14,3 173

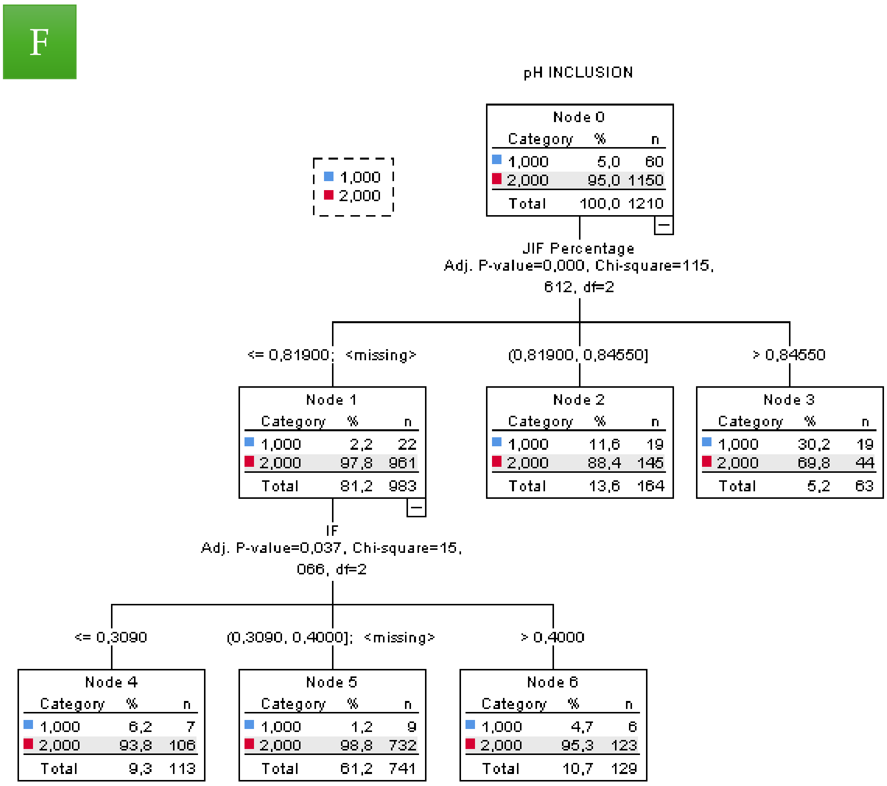
F
pH INCLUSION
1,000
2,000
Node 0
Category % n
1,000 5,0 60
2,000 95,0 1150
Total 100,0 1210
JIF Percentage
Adj. P-value=0,000, Chi-square=115,
612, df=2
<= 0,81900; <missing>
(0,81900, 0,84550]
> 0,84550
Node 1
Category % n
1,000 2,2 22
2,000 97,8 961
Total 81,2 983
Node 2
Category % n
1,000 11,6 19
2,000 88,4 145
Total 13,6 164
Node 3
Category % n
1,000 30,2 19
2,000 69,8 44
Total 5,2 63
IF
Adj. P-value=0,037, Chi-square=15,
066, df=2
<= 0,3090
(0,3090, 0,4000]; <missing>
> 0,4000
Node 4
Category % n
1,000 6,2 7
2,000 93,8 106
Total 9,3 113
Node 5
Category % n
1,000 1,2 9
2,000 98,8 732
Total 61,2 741
Node 6
Category % n
1,000 4,7 6
2,000 95,3 123
Total 10,7 129

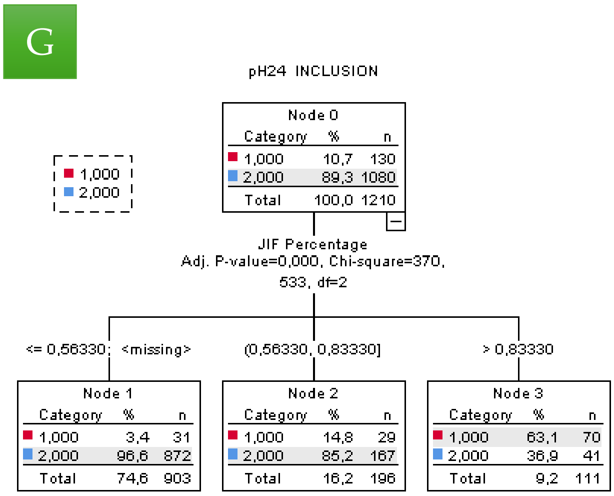
G
pH24 INCLUSION
1,000
2,000
Node 0
Category % n
1,000 10,7 130
2,000 89,3 1080
Total 100,0 1210
JIF Percentage
Adj. P-value=0,000, Chi-square=370,
533, df=2
<= 0,56330; <missing>
(0,56330, 0,83330]
> 0,83330
Node 1
Category % n
1,000 3,4 31
2,000 96,6 872
Total 74,6 903
Node 2
Category % n
1,000 14,8 29
2,000 85,2 167
Total 16,2 196
Node 3
Category % n
1,000 63,1 70
2,000 36,9 41
Total 9,2 111

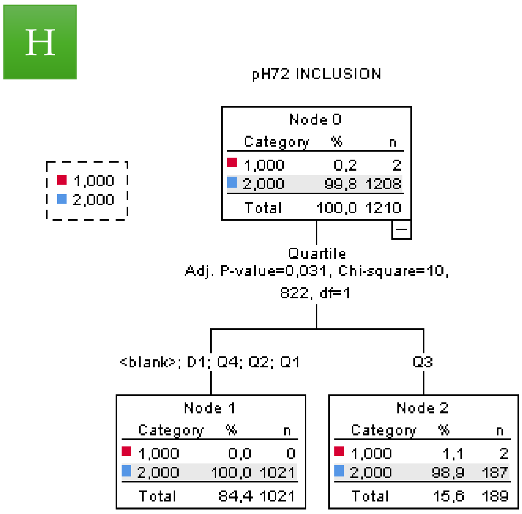
H
pH72 INCLUSION
1,000
2,000
Node 0
Category % n
1,000 0,2 2
2,000 99,8 1208
Total 100,0 1210
Quartile
Adj. P-value=0,031, Chi-square=10,
822, df=1
<blank>; D1; Q4; Q2; Q1
Q3
Node 1
Category % n
1,000 0,0 0
2,000 100,0 1021
Total 84,4 1021
Node 2
Category % n
1,000 1,1 2
2,000 98,9 187
Total 15,6 189

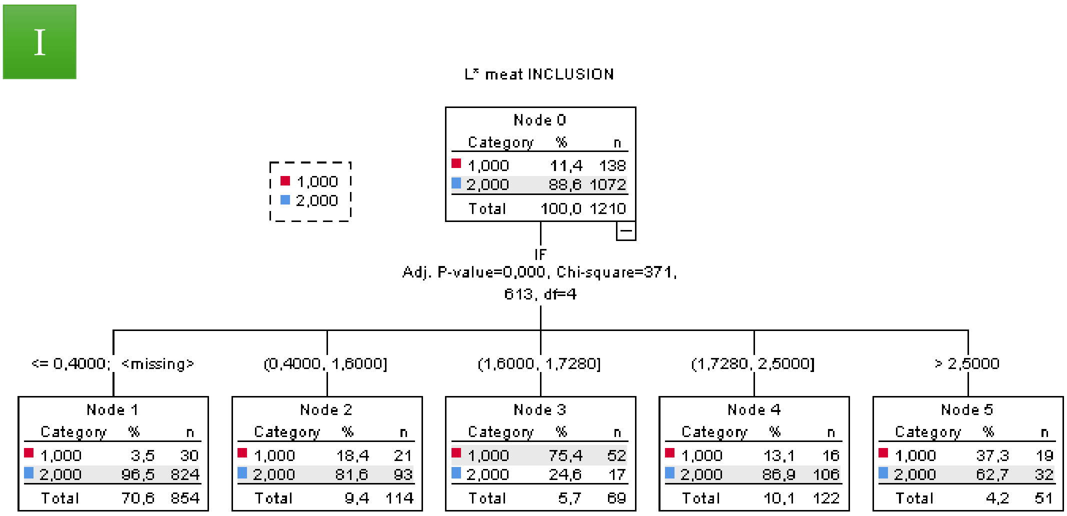
I
L* meat INCLUSION
1,000
2,000
Node 0
Category % n
1,000 11,4 138
2,000 88,6 1072
Total 100,0 1210
IF
Adj. P-value=0,000, Chi-square=371,
613, df=4
<= 0,4000; <missing>
(0,4000, 1,6000]
(1,6000, 1,7280]
(1,7280, 2,5000]
> 2,5000
Node 1
Category % n
1,000 3,5 30
2,000 96,5 824
Total 70,6 854
Node 2
Category % n
1,000 18,4 21
2,000 81,6 93
Total 9,4 114
Node 3
Category % n
1,000 75,4 52
2,000 24,6 17
Total 5,7 69
Node 4
Category % n
1,000 13,1 16
2,000 86,9 106
Total 10,1 122
Node 5
Category % n
1,000 37,3 19
2,000 62,7 32
Total 4,2 51

J

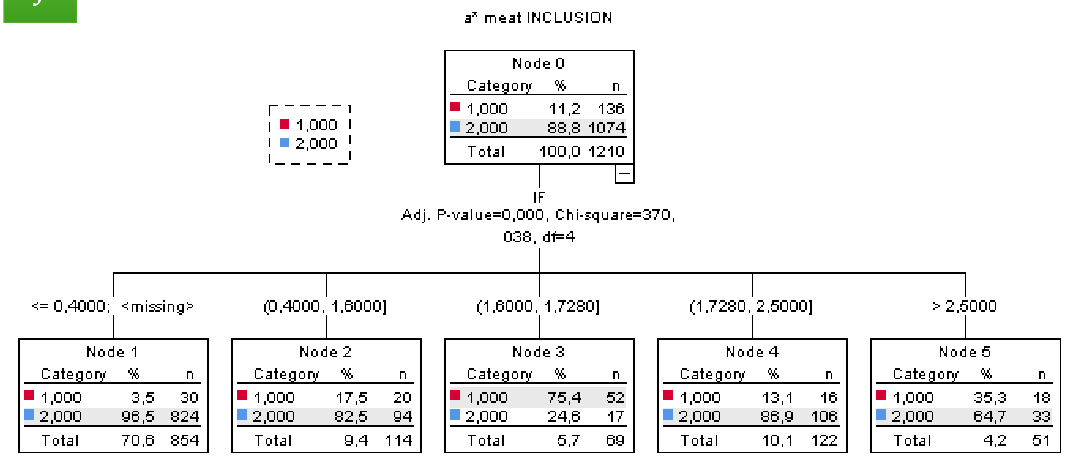
a* meat INCLUSION
1,000
2,000
Node 0
Category % n
1,000 11,2 136
2,000 88,8 1074
Total 100,0 1210
IF
Adj. P-value=0,000, Chi-square=370,
038, df=4
<= 0,4000; <missing>
(0,4000, 1,6000]
(1,6000, 1,7280]
(1,7280, 2,5000]
> 2,5000
Node 1
Category % n
1,000 3,5 30
2,000 96,5 824
Total 70,6 854
Node 2
Category % n
1,000 17,5 20
2,000 82,5 94
Total 9,4 114
Node 3
Category % n
1,000 75,4 52
2,000 24,6 17
Total 5,7 69
Node 4
Category % n
1,000 13,1 16
2,000 86,9 106
Total 10,1 122
Node 5
Category % n
1,000 35,3 18
2,000 64,7 33
Total 4,2 51

K

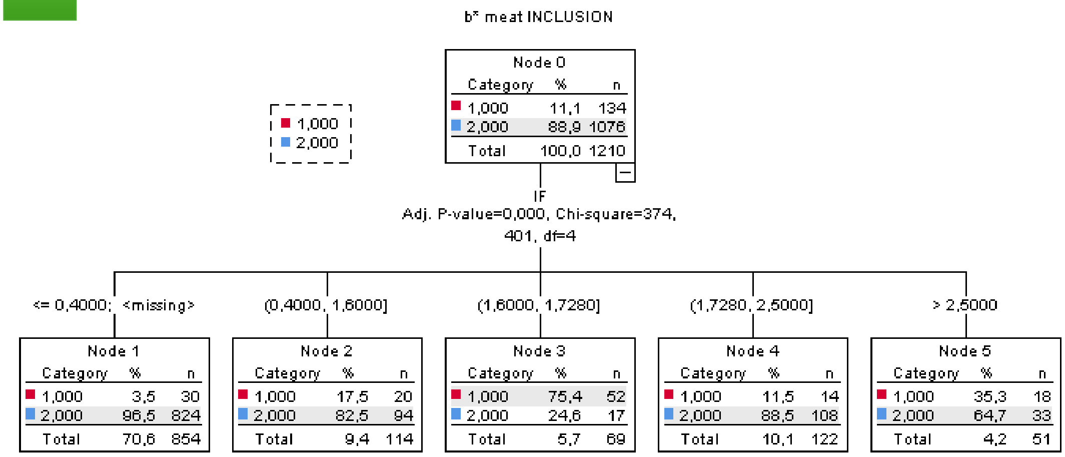
b* meat INCLUSION
1,000
2,000
Node 0
Category % n
1,000 11,1 134
2,000 88,9 1076
Total 100,0 1210
IF
Adj. P-value=0,000, Chi-square=374,
401, df=4
<= 0,4000; <missing>
(0,4000, 1,6000]
(1,6000, 1,7280]
(1,7280, 2,5000]
> 2,5000
Node 1
Category % n
1,000 3,5 30
2,000 96,5 824
Total 70,6 854
Node 2
Category % n
1,000 17,5 20
2,000 82,5 94
Total 9,4 114
Node 3
Category % n
1,000 75,4 52
2,000 24,6 17
Total 5,7 69
Node 4
Category % n
1,000 11,5 14
2,000 88,5 108
Total 10,1 122
Node 5
Category % n
1,000 35,3 18
2,000 64,7 33
Total 4,2 51

L

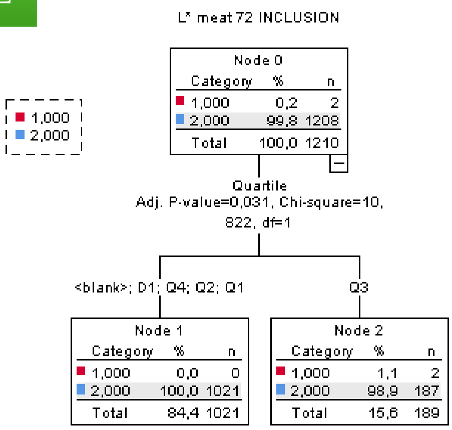
L* meat 72 INCLUSION
1,000
2,000
Node 0
Category % n
1,000 0,2 2
2,000 99,8 1208
Total 100,0 1210
Quartile
Adj. P-value=0,031, Chi-square=10,
822, df=1
<blank>; D1; Q4; Q2; Q1
Q3
Node 1
Category % n
1,000 0,0 0
2,000 100,0 1021
Total 84,4 1021
Node 2
Category % n
1,000 1,1 2
2,000 98,9 187
Total 15,6 189

M

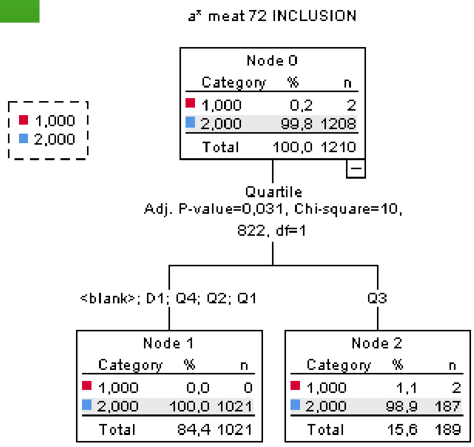

N **b* meat 72 inclusion:** only 1 of the 1210 observations belong to category 1, so the statistical program considers that this dependent variable is a constant.

O

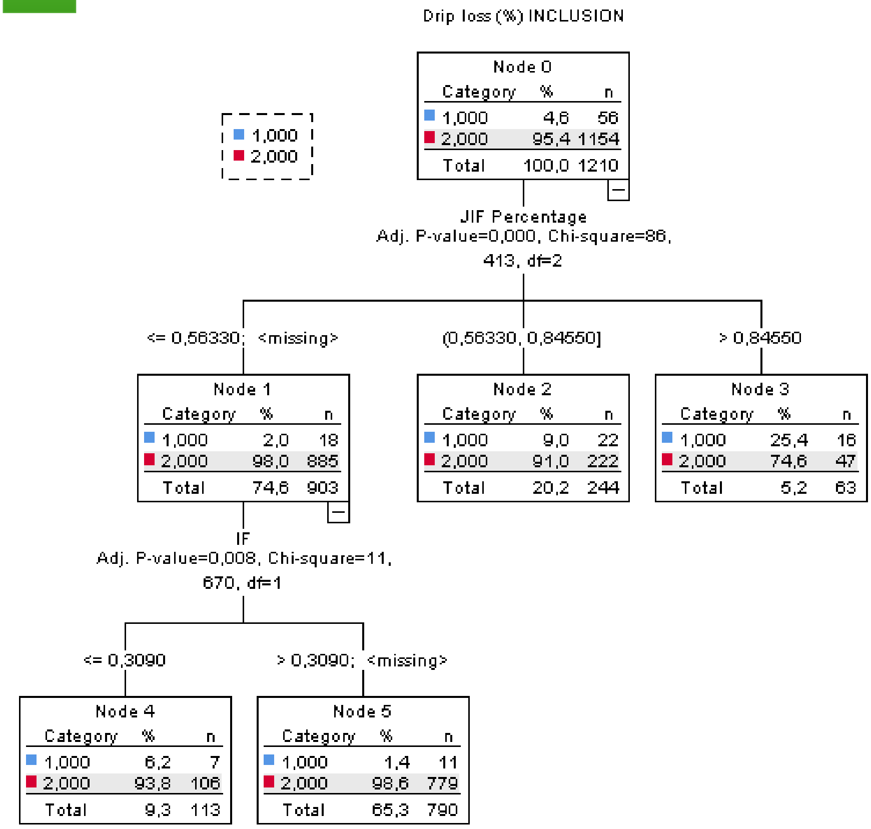

P

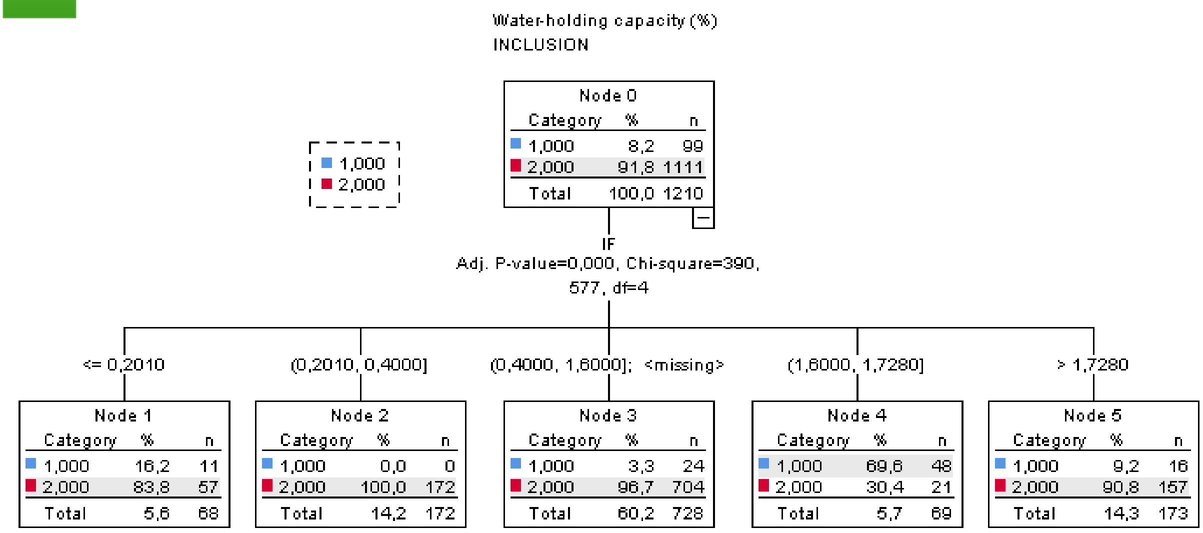
Water-holding capacity (%)
INCLUSION
1,000
2,000
Node 0
Category % n
1,000 8,2 99
2,000 91,8 1111
Total 100,0 1210
IF
Adj. P-value=0,000, Chi-square=390,
577, df=4
<= 0,2010
(0,2010, 0,4000]
(0,4000, 1,6000]; <missing>
(1,6000, 1,7280]
> 1,7280
Node 1
Category % n
1,000 16,2 11
2,000 83,8 57
Total 5,6 68
Node 2
Category % n
1,000 0,0 0
2,000 100,0 172
Total 14,2 172
Node 3
Category % n
1,000 3,3 24
2,000 96,7 704
Total 60,2 728
Node 4
Category % n
1,000 69,6 48
2,000 30,4 21
Total 5,7 69
Node 5
Category % n
1,000 9,2 16
2,000 90,8 157
Total 14,3 173

Q

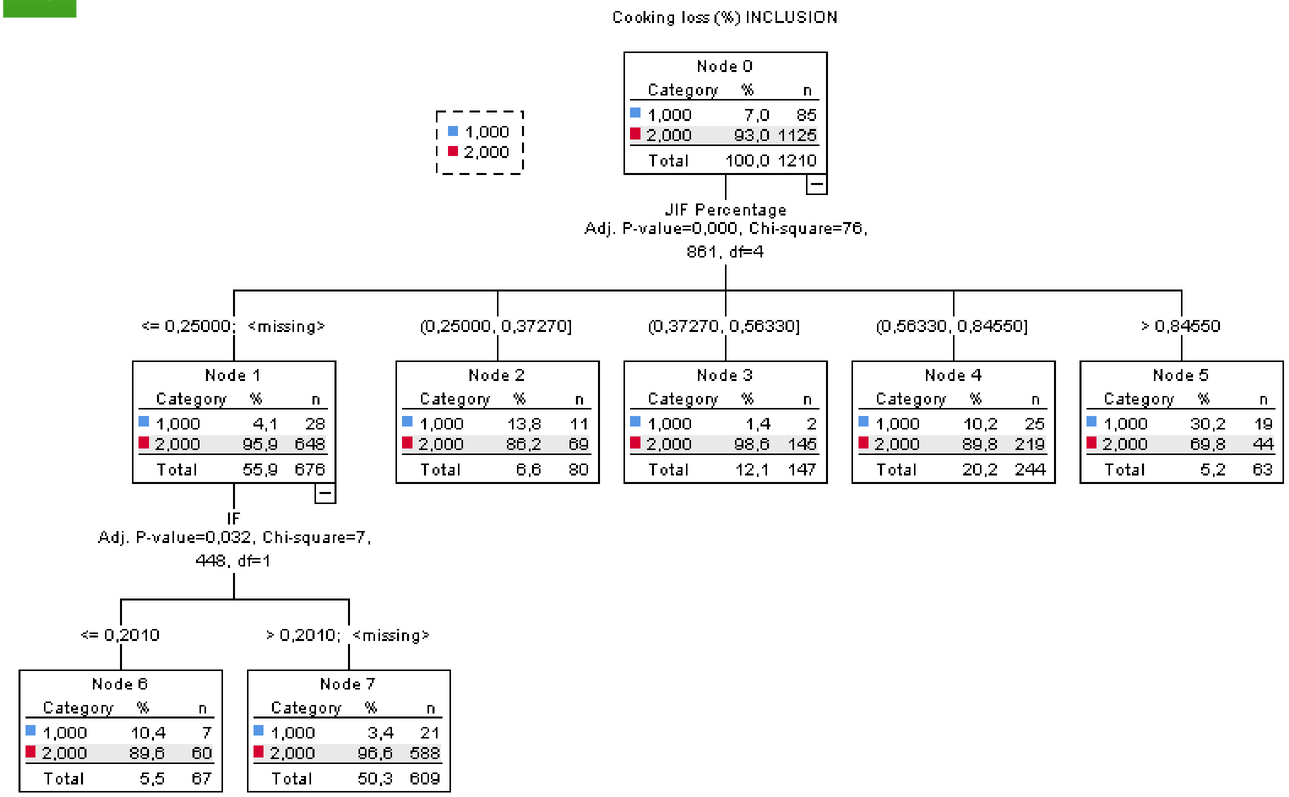
Cooking loss (%) INCLUSION
1,000
2,000
Node 0
Category % n
1,000 7,0 85
2,000 93,0 1125
Total 100,0 1210
JIF Percentage
Adj. P-value=0,000, Chi-square=76,
861, df=4
<= 0,25000; <missing>
(0,25000, 0,37270]
(0,37270, 0,56330]
(0,56330, 0,84550]
> 0,84550
Node 1
Category % n
1,000 4,1 28
2,000 95,9 648
Total 55,9 676
Node 2
Category % n
1,000 13,8 11
2,000 86,2 69
Total 6,6 80
Node 3
Category % n
1,000 1,4 2
2,000 98,6 145
Total 12,1 147
Node 4
Category % n
1,000 10,2 25
2,000 89,8 219
Total 20,2 244
Node 5
Category % n
1,000 30,2 19
2,000 69,8 44
Total 5,2 63
IF
Adj. P-value=0,032, Chi-square=7,
448, df=1
<= 0,2010
> 0,2010; <missing>
Node 6
Category % n
1,000 10,4 7
2,000 89,6 60
Total 5,5 67
Node 7
Category % n
1,000 3,4 21
2,000 96,6 588
Total 50,3 609

R

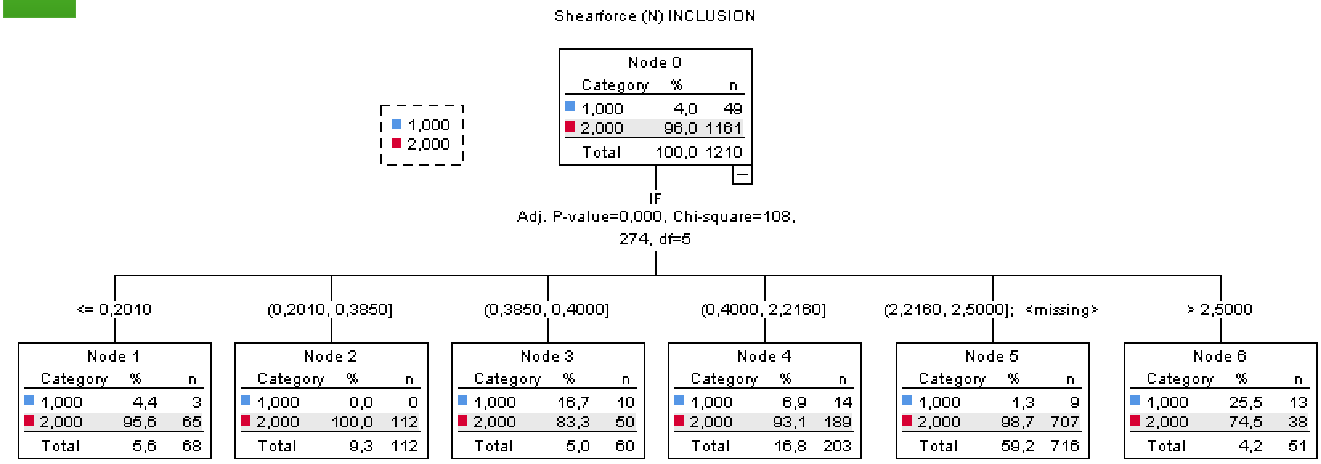
Shearforce (N) INCLUSION
1,000
2,000
Node 0
Category % n
1,000 4,0 49
2,000 96,0 1161
Total 100,0 1210
IF
Adj. P-value=0,000, Chi-square=108,
274, df=5
<= 0,2010
(0,2010, 0,3850]
(0,3850, 0,4000]
(0,4000, 2,2160]
(2,2160, 2,5000]; <missing>
> 2,5000
Node 1
Category % n
1,000 4,4 3
2,000 95,6 65
Total 5,6 68
Node 2
Category % n
1,000 0,0 0
2,000 100,0 112
Total 9,3 112
Node 3
Category % n
1,000 16,7 10
2,000 83,3 50
Total 5,0 60
Node 4
Category % n
1,000 6,9 14
2,000 93,1 189
Total 16,8 203
Node 5
Category % n
1,000 1,3 9
2,000 98,7 707
Total 59,2 716
Node 6
Category % n
1,000 25,5 13
2,000 74,5 38
Total 4,2 51

S

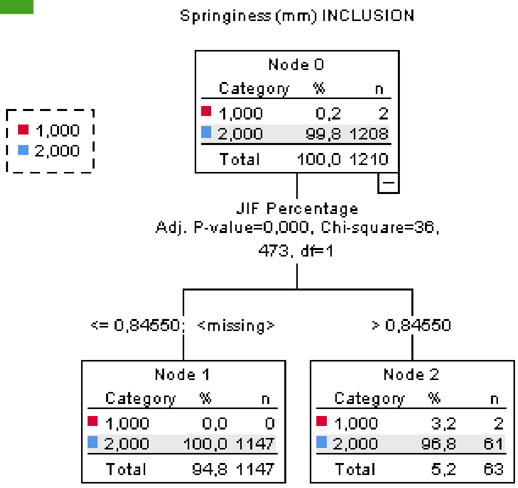
Springiness (mm) INCLUSION
1,000
2,000
Node 0
Category % n
1,000 0,2 2
2,000 99,8 1208
Total 100,0 1210
JIF Percentage
Adj. P-value=0,000, Chi-square=36,
473, df=1
<= 0,84550; <missing>
> 0,84550
Node 1
Category % n
1,000 0,0 0
2,000 100,0 1147
Total 94,8 1147
Node 2
Category % n
1,000 3,2 2
2,000 96,8 61
Total 5,2 63

T

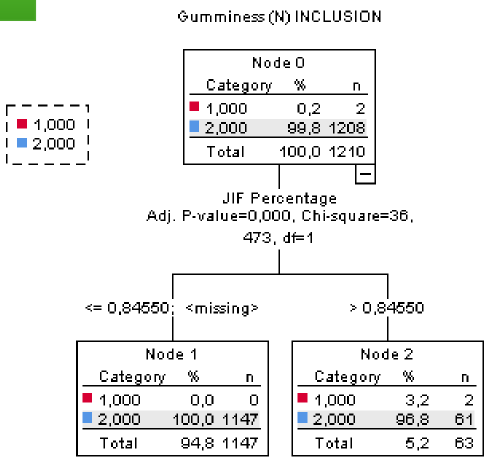
Gumminess (N) INCLUSION
1,000
2,000
Node 0
Category % n
1,000 0,2 2
2,000 99,8 1208
Total 100,0 1210
JIF Percentage
Adj. P-value=0,000, Chi-square=36,
473, df=1
<= 0,84550; <missing>
> 0,84550
Node 1
Category % n
1,000 0,0 0
2,000 100,0 1147
Total 94,8 1147
Node 2
Category % n
1,000 3,2 2
2,000 96,8 61
Total 5,2 63

U

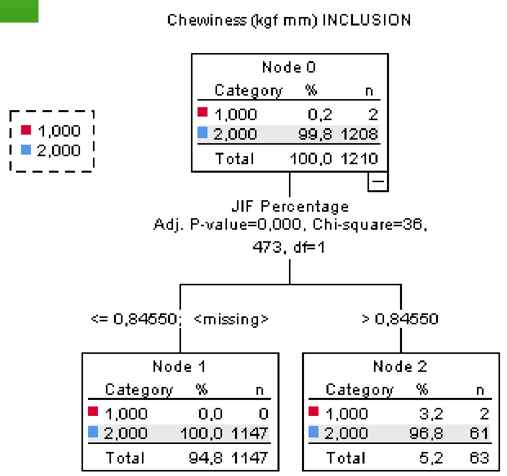

Chewiness (kgf mm) INCLUSION
1,000
2,000
Node 0
Category % n
1,000 0,2 2
2,000 99,8 1208
Total 100,0 1210
JIF Percentage
Adj. P-value=0,000, Chi-square=36,
473, df=1
<= 0,84550; <missing>
> 0,84550
Node 1
Category % n
1,000 0,0 0
2,000 100,0 1147
Total 94,8 1147
Node 2
Category % n
1,000 3,2 2
2,000 96,8 61
Total 5,2 63

V

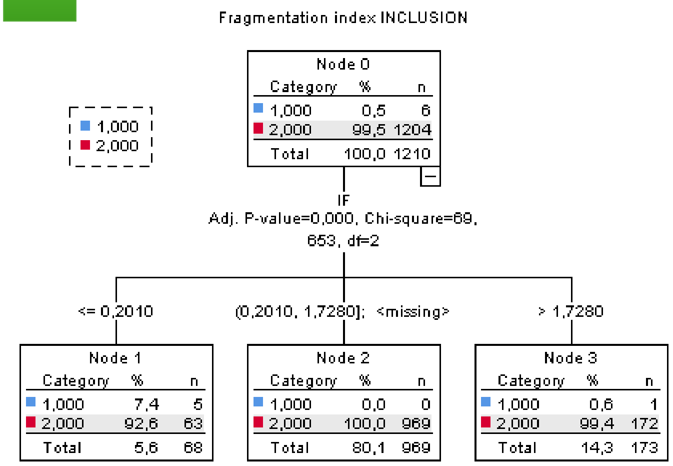

Fragmentation index INCLUSION
1,000
2,000
Node 0
Category % n
1,000 0,5 6
2,000 99,5 1204
Total 100,0 1210
IF
Adj. P-value=0,000, Chi-square=69,
653, df=2
<= 0,2010
(0,2010, 1,7280]; <missing>
> 1,7280
Node 1
Category % n
1,000 7,4 5
2,000 92,6 63
Total 5,6 68
Node 2
Category % n
1,000 0,0 0
2,000 100,0 969
Total 80,1 969
Node 3
Category % n
1,000 0,6 1
2,000 99,4 172
Total 14,3 173

W

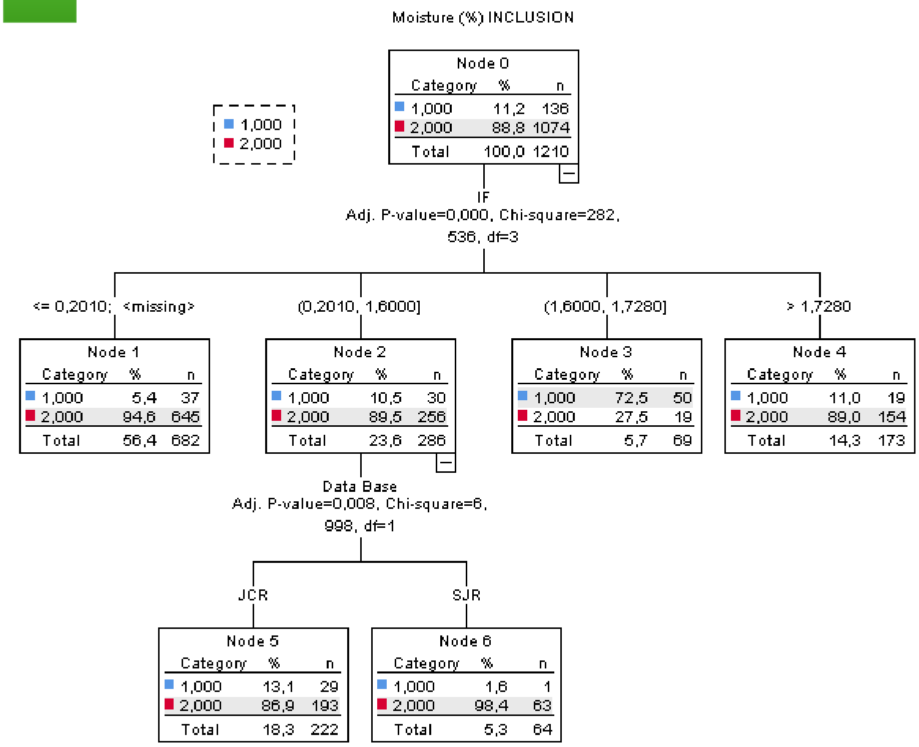
Moisture (%) INCLUSION
1,000
2,000
Node 0
Category % n
1,000 11,2 136
2,000 88,8 1074
Total 100,0 1210
IF
Adj. P-value=0,000, Chi-square=282, 536, df=3
<= 0,2010; <missing>
(0,2010, 1,6000]
(1,6000, 1,7280]
> 1,7280
Node 1
Category % n
1,000 5,4 37
2,000 94,6 645
Total 56,4 682
Node 2
Category % n
1,000 10,5 30
2,000 89,5 256
Total 23,6 286
Node 3
Category % n
1,000 72,5 50
2,000 27,5 19
Total 5,7 69
Node 4
Category % n
1,000 11,0 19
2,000 89,0 154
Total 14,3 173
Data Base
Adj. P-value=0,008, Chi-square=6, 998, df=1
JCR
SJR
Node 5
Category % n
1,000 13,1 29
2,000 86,9 193
Total 18,3 222
Node 6
Category % n
1,000 1,6 1
2,000 98,4 63
Total 5,3 64

X

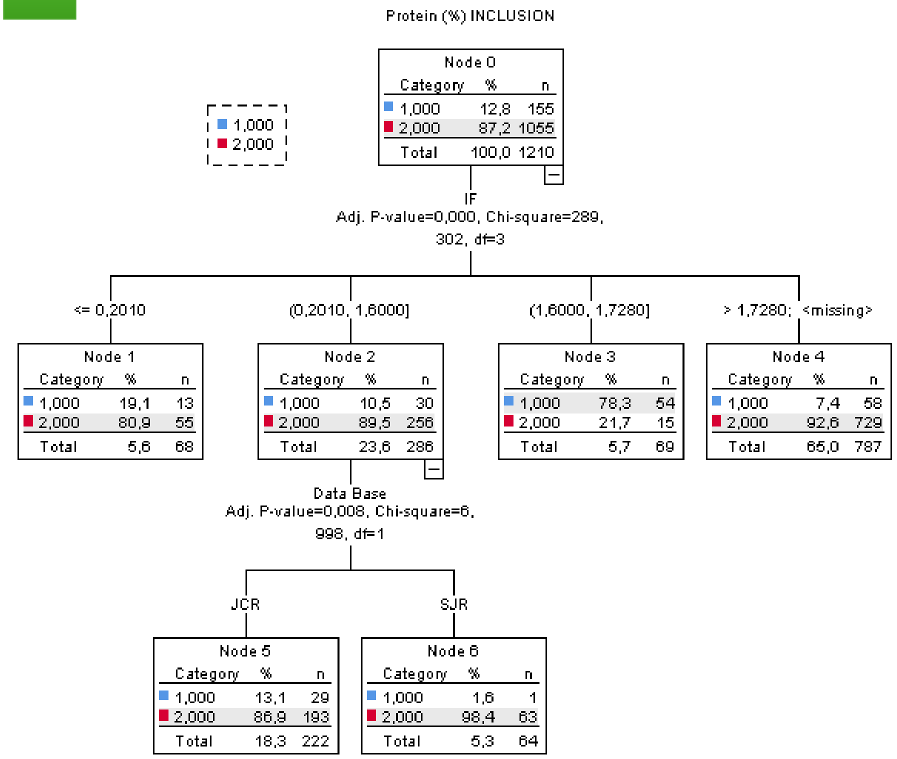
Protein (%) INCLUSION
1,000
2,000
Node 0
Category % n
1,000 12,8 155
2,000 87,2 1055
Total 100,0 1210
IF
Adj. P-value=0,000, Chi-square=289, 302, df=3
<= 0,2010
(0,2010, 1,6000]
(1,6000, 1,7280]
> 1,7280; <missing>
Node 1
Category % n
1,000 19,1 13
2,000 80,9 55
Total 5,6 68
Node 2
Category % n
1,000 10,5 30
2,000 89,5 256
Total 23,6 286
Node 3
Category % n
1,000 78,3 54
2,000 21,7 15
Total 5,7 69
Node 4
Category % n
1,000 7,4 58
2,000 92,6 729
Total 65,0 787
Data Base
Adj. P-value=0,008, Chi-square=6, 998, df=1
JCR
SJR
Node 5
Category % n
1,000 13,1 29
2,000 86,9 193
Total 18,3 222
Node 6
Category % n
1,000 1,6 1
2,000 98,4 63
Total 5,3 64

Y

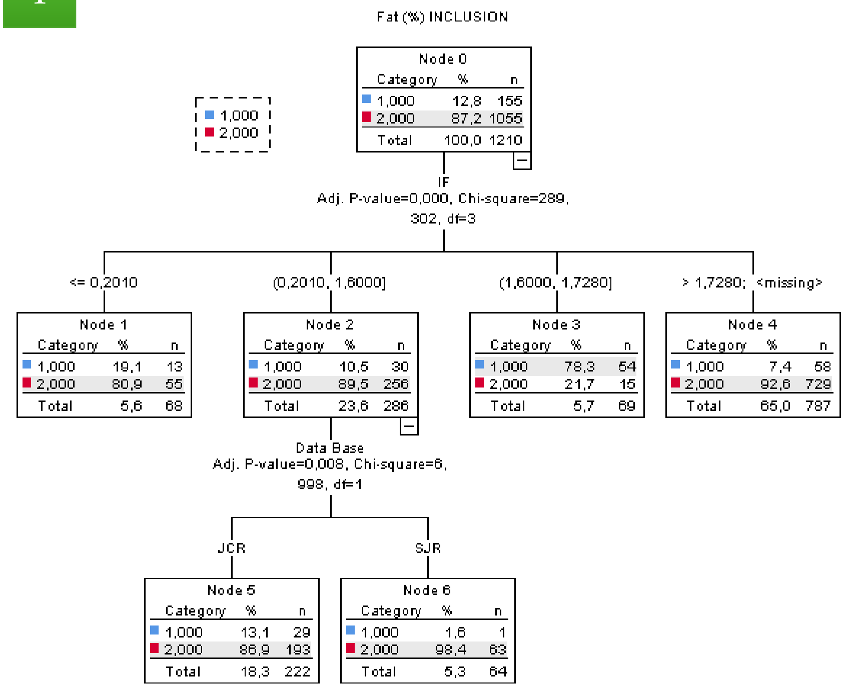
Fat (%) INCLUSION
1,000
2,000
Node 0
Category % n
1,000 12,8 155
2,000 87,2 1055
Total 100,0 1210
IF
Adj. P-value=0,000, Chi-square=289,
302, df=3
<= 0,2010
(0,2010, 1,6000]
(1,6000, 1,7280]
> 1,7280; <missing>
Node 1
Category % n
1,000 19,1 13
2,000 80,9 55
Total 5,6 68
Node 2
Category % n
1,000 10,5 30
2,000 89,5 256
Total 23,6 286
Node 3
Category % n
1,000 78,3 54
2,000 21,7 15
Total 5,7 69
Node 4
Category % n
1,000 7,4 58
2,000 92,6 729
Total 65,0 787
Data Base
Adj. P-value=0,008, Chi-square=6,
998, df=1
JCR
SJR
Node 5
Category % n
1,000 13,1 29
2,000 86,9 193
Total 18,3 222
Node 6
Category % n
1,000 1,6 1
2,000 98,4 63
Total 5,3 64

Z

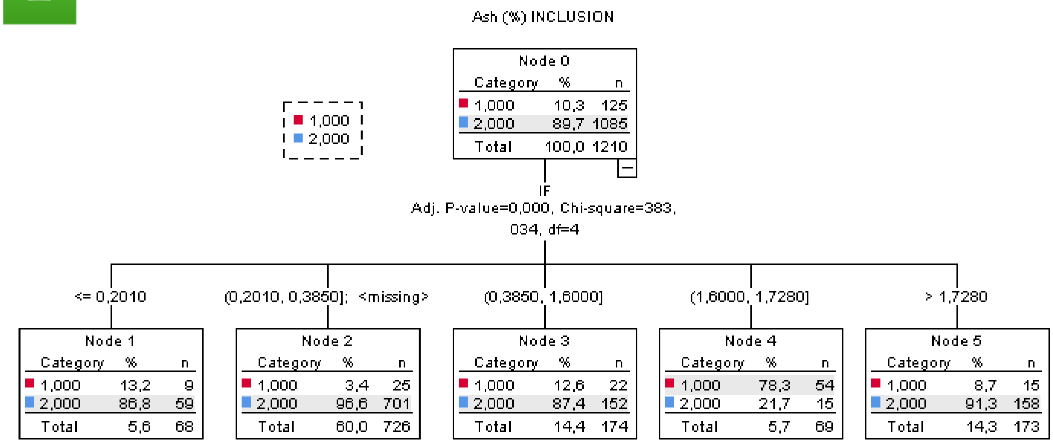
Ash (%) INCLUSION
1,000
2,000
Node 0
Category % n
1,000 10,3 125
2,000 89,7 1085
Total 100,0 1210
IF
Adj. P-value=0,000, Chi-square=383,
034, df=4
<= 0,2010
(0,2010, 0,3850]; <missing>
(0,3850, 1,6000]
(1,6000, 1,7280]
> 1,7280
Node 1
Category % n
1,000 13,2 9
2,000 86,8 59
Total 5,6 68
Node 2
Category % n
1,000 3,4 25
2,000 96,6 701
Total 60,0 726
Node 3
Category % n
1,000 12,6 22
2,000 87,4 152
Total 14,4 174
Node 4
Category % n
1,000 78,3 54
2,000 21,7 15
Total 5,7 69
Node 5
Category % n
1,000 8,7 15
2,000 91,3 158
Total 14,3 173

AA

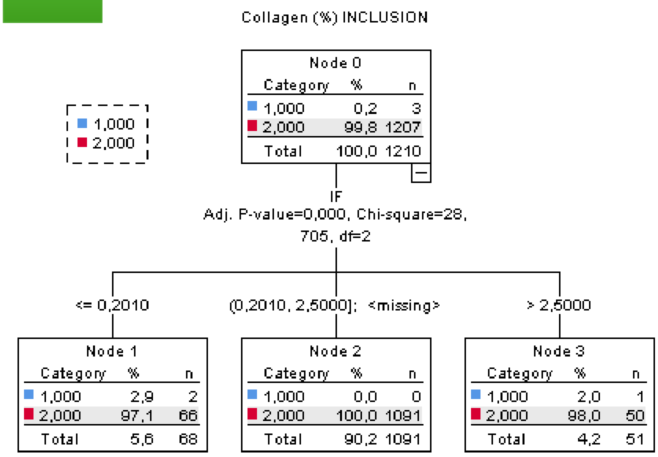
Collagen (%) INCLUSION
1,000
2,000
Node 0
Category % n
1,000 0,2 3
2,000 99,8 1207
Total 100,0 1210
IF
Adj. P-value=0,000, Chi-square=28,
705, df=2
<= 0,2010
(0,2010, 2,5000]; <missing>
> 2,5000
Node 1
Category % n
1,000 2,9 2
2,000 97,1 66
Total 5,6 68
Node 2
Category % n
1,000 0,0 0
2,000 100,0 1091
Total 90,2 1091
Node 3
Category % n
1,000 2,0 1
2,000 98,0 50
Total 4,2 51

AB

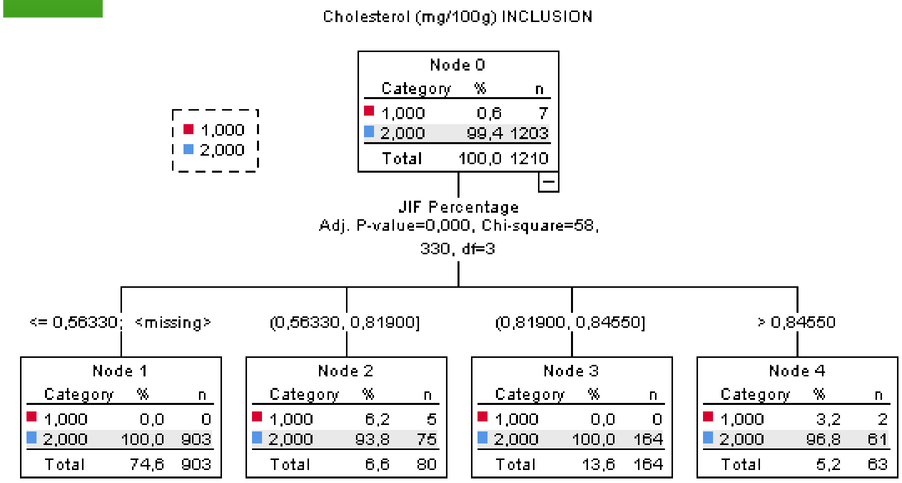
Cholesterol (mg/100g) INCLUSION
1,000
2,000
Node 0
Category % n
1,000 0,6 7
2,000 99,4 1203
Total 100,0 1210
JIF Percentage
Adj. P-value=0,000, Chi-square=58,
330, df=3
<= 0,56330; <missing>
(0,56330, 0,81900]
(0,81900, 0,84550]
> 0,84550
Node 1
Category % n
1,000 0,0 0
2,000 100,0 903
Total 74,6 903
Node 2
Category % n
1,000 6,2 5
2,000 93,8 75
Total 6,6 80
Node 3
Category % n
1,000 0,0 0
2,000 100,0 164
Total 13,6 164
Node 4
Category % n
1,000 3,2 2
2,000 96,8 61
Total 5,2 63
