# Supplementary material for: Data-Mining Methodology to Improve the Scientific Production Quality in Turkey Meat and Carcass Characterization Studies
Source: Animals (Basel). 2024 Jul 19;14(14):2107. doi: 10.3390/ani14142107 (PMC11273658; doi:10.3390/ani14142107)
Supplement: Supplementary file 1 [file animals-14-02107-s001.zip › animals-3088178-supplementary/Supplementary Table S1.pdf]

**Supplementary Table S1.** Values for risk estimates and standard errors for both methods applied in the cross-validation test for each tree.

| Variable | Method | Risk Estimate | Std. Error |
|---|---|---|---|
| Carcass/piece weight | Resubstitution | 0.302 | 0.013 |
| | Cross-Validation | 0.302 | 0.013 |
| Carcass/piece yield | Resubstitution | 0.229 | 0.012 |
| | Cross-Validation | 0.229 | 0.012 |
| Cold canal weight | Resubstitution | 0.136 | 0.010 |
| | Cross-Validation | 0.136 | 0.010 |
| Slaughter weight | Resubstitution | 0.175 | 0.011 |
| | Cross-Validation | 0.175 | 0.011 |
| Muscle fiber diameter | Resubstitution | 0.003 | 0.002 |
| | Cross-Validation | 0.003 | 0.002 |
| pH | Resubstitution | 0.050 | 0.006 |
| | Cross-Validation | 0.050 | 0.006 |
| pH 24h | Resubstitution | 0.083 | 0.008 |
| | Cross-Validation | 0.083 | 0.008 |
| pH 72h | Resubstitution | 0.002 | 0.001 |
| | Cross-Validation | 0.002 | 0.001 |
| L* meat | Resubstitution | 0.085 | 0.008 |
| | Cross-Validation | 0.085 | 0.008 |
| a* meat | Resubstitution | 0.083 | 0.008 |
| | Cross-Validation | 0.083 | 0.008 |
| b* meat | Resubstitution | 0.082 | 0.008 |
| | Cross-Validation | 0.082 | 0.008 |
| L* meat 72h | Resubstitution | 0.002 | 0.001 |
| | Cross-Validation | 0.002 | 0.001 |
| a* meat 72h | Resubstitution | 0.002 | 0.001 |
| | Cross-Validation | 0.002 | 0.001 |
| b* meat 72h | Resubstitution | - | - |
| | Cross-Validation | - | - |
| Drip loss | Resubstitution | 0.046 | 0.006 |
| | Cross-Validation | 0.046 | 0.006 |
| Water-holding capacity | Resubstitution | 0.060 | 0.007 |
| | Cross-Validation | 0.060 | 0.007 |
| Cooking loss | Resubstitution | 0.070 | 0.007 |
| | Cross-Validation | 0.070 | 0.007 |
| Shear force | Resubstitution | 0.040 | 0.006 |
| | Cross-Validation | 0.040 | 0.006 |

| | | | |
|---|---|---|---|
| Springiness | Resubstitution | 0.002 | 0.001 |
| | Cross-Validation | 0.002 | 0.001 |
| Gumminess | Resubstitution | 0.002 | 0.001 |
| | Cross-Validation | 0.002 | 0.001 |
| Chewiness | Resubstitution | 0.002 | 0.001 |
| | Cross-Validation | 0.002 | 0.001 |
| Fragmentation index | Resubstitution | 0.005 | 0.002 |
| | Cross-Validation | 0.005 | 0.002 |
| Moisture | Resubstitution | 0.087 | 0.008 |
| | Cross-Validation | 0.087 | 0.008 |
| Protein | Resubstitution | 0.096 | 0.008 |
| | Cross-Validation | 0.096 | 0.008 |
| Fat | Resubstitution | 0.096 | 0.008 |
| | Cross-Validation | 0.096 | 0.008 |
| Ash | Resubstitution | 0.071 | 0.07 |
| | Cross-Validation | 0.071 | 0.07 |
| Collagen | Resubstitution | 0.002 | 0.001 |
| | Cross-Validation | 0.002 | 0.001 |
| Cholesterol | Resubstitution | 0.006 | 0.002 |
| | Cross-Validation | 0.006 | 0.002 |
